# The Putative Bromodomain Protein PfBDP7 of the Human Malaria Parasite *Plasmodium Falciparum* Cooperates With PfBDP1 in the Silencing of Variant Surface Antigen Expression

**DOI:** 10.3389/fcell.2022.816558

**Published:** 2022-04-12

**Authors:** Jennifer E. Quinn, Myriam D. Jeninga, Katharina Limm, Kapil Pareek, Tina Meißgeier, Anna Bachmann, Michael F. Duffy, Michaela Petter

**Affiliations:** ^1^ Mikrobiologisches Institut—Klinische Mikrobiologie, Immunologie und Hygiene, Universitätsklinikum Erlangen, Friedrich-Alexander-Universität (FAU) Erlangen-Nürnberg, Erlangen, Germany; ^2^ Institute of Functional Genomics, University of Regensburg, Regensburg, Germany; ^3^ Department of Cellular Parasitology, Bernhard Nocht Institute for Tropical Medicine, Hamburg, Germany; ^4^ Centre for Structural Systems Biology (CSSB), Hamburg, Germany; ^5^ Biology Department, University of Hamburg, Hamburg, Germany; ^6^ Department of Microbiology and Immunology, The University of Melbourne, Bio21 Institute, Parkville, VIC, Australia; ^7^ Department of Medicine, The University of Melbourne, Royal Melbourne Hospital, Parkville, VIC, Australia

**Keywords:** malaria, *P. falciparum*, chromatin, bromodomain, variant surface antigens

## Abstract

Epigenetic regulation is a critical mechanism in controlling virulence, differentiation, and survival of the human malaria parasite *Plasmodium (P.) falciparum*. Bromodomain proteins contribute to this process by binding to acetylated lysine residues of histones and thereby targeting the gene regulatory machinery to gene promoters. A protein complex containing the *P. falciparum* bromodomain proteins (PfBDP) 1 and PfBDP2 (BDP1/BDP2 core complex) was previously shown to play an essential role for the correct transcription of invasion related genes. Here, we performed a functional characterization of a third component of this complex, which we dubbed PfBDP7, because structural modelling predicted a typical bromodomain fold. We confirmed that PfBDP7 is a nuclear protein that interacts with PfBDP1 at invasion gene promoters in mature schizont stage parasites and contributes to their transcription. Although partial depletion of PfBDP7 showed no significant effect on parasite viability, conditional knock down of either PfBDP7 or PfBDP1 resulted in the de-repression of variant surface antigens (VSA), which are important pathogenicity factors. This de-repression was evident both on mRNA and protein level. To understand the underlying mechanism, we mapped the genome wide binding sites of PfBDP7 by ChIPseq and showed that in early schizonts, PfBDP7 and PfBDP1 are commonly enriched in heterochromatic regions across the gene body of all VSA families, including genes coding for PfEMP1, RIFIN, STEVOR, and PfMC-2TM. This suggests that PfBDP7 and PfBDP1 contribute to the silencing of VSAs by associating with heterochromatin. In conclusion, we identified PfBDP7 as a chromatin binding protein that is a constitutive part of the *P. falciparum* BDP1/BDP2 core complex and established PfBDP1 and PfBDP7 as novel players in the silencing of heterochromatin regulated virulence gene families of the malaria parasite *P. falciparum*.

## Introduction

Malaria, in particular caused by the apicomplexan parasite *Plasmodium (P.) falciparum,* remains a major health concern in many poverty-stricken countries of the world. In 2019, malaria infections resulted in an estimated 409.000 deaths, of which 67% occurred in children under the age of 5 years (WHO, 2020). Recent achievements in lowering the burden of malaria are threatened by the COVID-19 pandemic and the limited availability of malaria control measures in the countries where they are most needed. Mathematical models predict that malaria mortality might significantly increase in the years ahead (Weiss et al., 2021).

*P. falciparum* is transmitted to humans by the bite of a female *Anopheles* mosquito. The transmitted sporozoites initially infect hepatocytes, in which they massively replicate by schizogony, before the progeny liver merozoites enter the blood stream, where they establish a continuous cycle of erythrocyte infection. This intra-erythrocytic phase is responsible for the different clinical manifestation of the malaria disease, which can range from asymptomatic or mild courses to life threatening complications including severe anemia, cerebral malaria, or metabolic acidosis (Miller et al., 2013). A key mechanism underlying the pathogenesis of *P. falciparum* malaria is the expression of adhesive parasite-derived proteins on the surface of infected host cells. These proteins belong to several variant surface antigen (VSA) families, which are encoded in multi-gene families in the *P. falciparum* genome and include the *var*, *rif*, *stevor* and *pfmc-2tm* families (Smith et al., 1995; Su et al., 1995; Cheng et al., 1998; Kyes et al., 1999; Sam-Yellowe et al., 2004; Bachmann et al., 2015). The interaction of VSAs with receptors on host cells contributes in multiple ways to the disease, depending both on the protein family as well as on the particular variant involved. *Var* gene-encoded PfEMP1 (*P. falciparum* erythrocyte membrane protein 1) variants can bind to a range of host cell receptors expressed on the endothelium, causing sequestration of the infected cells in small capillaries and contributing to tissue damage by endothelial activation and blood vessel occlusion (Miller et al., 2013). RIFINs, which are encoded by about 150 different *rif* (repetitive interspersed family) gene variants per parasite genome, have been implicated in the binding of non-infected erythrocytes by an infected erythrocyte, a phenomenon referred to as rosetting (Fernandez et al., 1999; Goel et al., 2015). In addition, several recent studies have demonstrated that certain RIFIN variants associated with severe disease can impair the human immune response by engaging LILRB1, LILRB2 and LAIR1 inhibitory receptors on natural killer cells and B-cells (Saito et al., 2017; Sakoguchi et al., 2021). STEVORs (subtelomeric variable open reading frame) contribute to rosetting and host cell invasion (Niang et al., 2014; Wichers et al., 2019) and modulate membrane deformability in asexual and gametocyte infected RBCs (Sanyal et al., 2012; Naissant et al., 2016). PfMC-2TM (*P. falciparum* Maurer’s Clefts 2 transmembrane) family proteins are also exported to the erythrocyte membrane (Bachmann et al., 2012; Bachmann et al., 2015); however, their biological function remains speculative.

The genes coding for VSAs are not randomly distributed along the 14 chromosomes of *P. falciparum*, but are organized in clusters in the subtelomeric regions and in a few central locations (Gardner et al., 2002). In a single parasite, only one or a few variants of each family are actively transcribed, while the majority of genes is silenced by packaging into a heterochromatic structure that relies on methylation of histone H3 on lysine 9 (H3K9me3) and heterochromatin protein 1 (HP1), which binds to this modification (Flueck et al., 2009; Lopez-Rubio et al., 2009). Several histone modifying proteins have been implicated in the modulation of the chromatin structure at the subtelomeric and central VSA clusters and, consequently, the regulation of VSA expression. Active histone deacetylation is critical for the repression of the genes coding for VSA families, as knock out of the sirtuin histone deacetylases Sir2A and Sir2B (Duraisingh et al., 2005; Freitas-Junior et al., 2005; Tonkin et al., 2009) or conditional depletion of histone deacetylase 2, PfHda2 (Coleman et al., 2014), results in de-repression of subtelomeric and central *var* genes. The methyltransferase PfSET2/PfSETvs contributes to *var* gene silencing by mediating H3K36me3 at *var* gene bodies (Jiang et al., 2013) and has been implicated in the maintenance of mutually exclusive *var* gene expression (Ukaegbu et al., 2014). In their active form, *var* genes lose the H3K9me3 modification, particularly along the promoter, and instead acquire the histone variants H2A.Z and H2B.Z (Petter et al., 2011; Petter et al., 2013) as well as several histone acetylation marks (H3K27ac, H3K9ac, H3K18ac) and H3K4me3 (Lopez-Rubio et al., 2007; Duffy et al., 2017). During progression through the life cycle, the active *var* gene is transiently repressed coinciding with a partial loss of the activating marks. The histone methyltransferase PfSET10 has been linked to this process by methylation of H3K4me2, which is enriched in schizonts at *var* genes that are poised for activation in the next parasite replication cycle (Lopez-Rubio et al., 2007; Volz et al., 2012). These studies show that the dynamic chromatin landscape at VSA genes is critical for controlling the virulence gene expression program. However, it is still poorly understood, how the histone modification cues are mechanistically linked to *var* gene transcription and how transcription of other virulence gene families is regulated.

Bromodomain proteins bind to acetylated lysine residues on histones and interact with specific transcription factors to recruit the chromatin remodeling and transcriptional machineries (Josling et al., 2012; Fujisawa and Filippakopoulos, 2017). In *P. falciparum*, eight bromodomain proteins have been predicted [PfBDP1-4, PfTAF1 (PfBDP5), PfTAF2 (PfBDP6), PfSET1 and PfGCN5 (Nguyen et al., 2020)], of which only PfBDP1 and the histone acetyltransferase PfGCN5 have been functionally characterized (Cui et al., 2007; Josling et al., 2015; Bhowmick et al., 2020; Rawat et al., 2020; Miao et al., 2021; Rawat et al., 2021). PfBDP1 is an essential protein that binds to acetylated histones notably in actively transcribed gene promoter regions (Josling et al., 2015). Through its interaction with the transcription factor ApiAP2-I in schizont stage parasites, PfBDP1 coordinates the expression of genes with a functional role in erythrocyte invasion (Santos et al., 2017). PfBDP1 is part of a multi-protein complex, which binds to various acetylated histones including H2A.Z, H2B.Z, H3 and H4. The complex was named “BDP1/BDP2-core complex” due to the presence of another bromodomain protein, PfBDP2 (Josling et al., 2015; Toenhake et al., 2018; Hoeijmakers et al., 2019). Interestingly, ApiAP2-I recruitment seems to depend on the interaction of the complex with acetylated H2B.Z, whereas acetylation of H4 was not sufficient to recruit ApiAP2-I. This suggests that bromodomain proteins exist in functionally distinct sub-complexes, which, depending on the chromatin context, fine-tune gene expression due to the combinatorial action of components with different binding specificities (Hoeijmakers et al., 2019).

Several other proteins were variably detected to be associated with the BDP1/BDP2 core complex. One candidate which was consistently enriched in several studies is a “conserved protein of unknown function” with the accession number Pf3D7_1124300 (Josling et al., 2015; Toenhake et al., 2018; Hoeijmakers et al., 2019). Here, we aimed to investigate the molecular function of Pf3D7_1124300 and its association with PfBDP1 to gain a better understanding of the molecular mechanisms underlying gene regulation in *P. falciparum.*

## Materials and Methods

### Sequence Analysis

Sequences of *P. falciparum* bromodomain proteins were retrieved from PlasmoDB.org. Accession numbers are PfBDP7: PF3D7_1124300, PfGCN5: PF3D7_0823300, PfSET1: PF3D7_0629700, PfBDP1: PF3D7_1033700, PfBDP2: PF3D7_1212900, PfBDP3: PF3D7_0110500, PfBDP4: PF3D7_1475600, PfBDP5/TAF1: PF3D7_1234100. Orthologs of PfBDP7 were searched on http://www.veupathdb.org. Multiple pairwise alignments were performed using ClustalW embedded in Geneious Prime 2019 (Biomatters). The full length PfBDP7 protein sequence was submitted to the online protein structure prediction tool Phyre2 (Kelley et al., 2015) and visualized using First Glance in Jmol (http://firstglance.jmol.org).

### *P. falciparum* Lines and Continuous Culture

*P. falciparum* parasites of the strains 3D7 or NF54 and the transgenic derivatives were cultivated in O+ or A+ erythrocytes (purchased from the Bavarian Red Cross Service) in RPMI 1640 medium supplemented with 0.25% Albumax II and 5% human AB+ serum (purchased from the Bavarian Red Cross Service). Cultures were kept in a hypoxic environment of 1% oxygen at 37°C. Parasitemia was monitored daily by Giemsa smear and cultures were synchronized with 5% sorbitol on a regular basis. For tight synchronization to an 8 h window, parasites from cultures containing mainly segmented schizonts were enriched by MACS (VarioMACS, CS columns, Miltenyi) and sorbitol treatment was conducted 8 h later to remove schizonts which had not yet invaded.

### Plasmid Construction, Transfection and Screening for Plasmid Integration

*P. falciparum* DNA fragments were amplified from genomic DNA of the 3D7 strain with primers specified in Supplementary Table S1. For the transfection plasmids pSLI_PfBDP7TyGlmS pSLI_PfBDP7BirATy, PCR fragments representing a 937 bp fragment at the 3′ end of PfBDP7 were amplified with primers 443 and 451 and cloned into the plasmid pGCN5_5xTy using the XmaI and NheI sites (replacing the GCN5 flank in that plasmid). The 2A-neomycin resistance cassette (2A Neo) was amplified from pSLI-TGD (Birnbaum et al., 2017) using primers 484 and 485 and inserted into the SpeI site 3′ of the PfBDP7 flank. For pSLI_PfBDP7TyGlmS, a GlmS sequence (Prommana et al., 2013) was amplified from pTEX150_HA_Glms 2_Linker (Elsworth et al., 2014) and integrated into the newly generated MluI and AvrII sites 3′ of 2ANeo. For pSLI_PfBDP7BirATy, the BirA* sequence was amplified from pH-BirA-GFP and inserted upstream of the 5xTy tags using the NheI sites. Finally, the hDHFR sequence was exchanged for a BSD sequence derived from pSkipFlox (Birnbaum et al., 2017) using the EcoRI and EcoRV sites. Correct integration was verified by Sanger sequencing after each cloning step (Macrogen). 100 µg of plasmid was used for transfection of the 3D7 or 3D7::PfBDP1HA (Josling et al., 2015) parasite lines and selection for episomal plasmids was conducted using 2.5 nM WR99210 (for 3D7::PfBDP7TyGlmS) or 2.5 μg/ml Blasticidin S (for PfBDP1HA::PfBDP7BirATy). After retrieval of resistant parasites, parasites were further selected for presence of the PfBDP7Ty2ANeo or PfBDP7BirATy2ANeo fusion genes using 400 μg/ml G418 (Carl Roth). Correct integration into the PfBDP7 locus was verified by diagnostic PCR using primers positioned outside of the cloned flank to amplify the wild type (wt) locus (wt: 587 and 588) and by combinations of primers targeting sequences in the wt locus with primers targeting the plasmid (5′ integration: 587 and 684, 3′ integration: 686 and 588). The presence of the plasmid was verified using primers 684 and 686, and the *pfs16* gene was amplified as a positive control. NF54::PfBDP1HA parasites were generated by transfection of NF54 parasites with the plasmid pSLI_PfBDP1HA, in which a 3’ homology flank of PfBDP1 was fused to 3xHA tags.

### Parasite Proliferation Assays

Relative parasite proliferation was determined either using a SYBR Green I-based fluorescence assay (Leidenberger et al., 2017) or by flow cytometry. For flow cytometry, cultures were fixed in PBS containing 4% formaldehyde and 0.0075% glutaraldehyde and stained with 20 µM Hoechst 33,342 (Invitrogen) and 1 μg/ml thiazole orange (Sigma Aldrich) for 30 min. Parasites were washed twice with PBS before acquisition on a BD FACSCanto II cytometer. Data were analyzed using the FlowJo software. For the SYBR Green I based assay, parasite cultures were lysed in lysis buffer [20 mM Tris pH 7.5, 5 mM EDTA, 0.008% saponin, 0.08% Triton X-100, 0.1 μg/ml SYBR Green I (Invitrogen)] and incubated for 30 min before measuring the fluorescence signal on a ThermoScan microplate reader at 485 nm excitation and 535 nm emission wavelength.

### Immunofluorescence Microscopy

Smears of parasite cultures on glass slides were fixed in ice cold 10% methanol/90% acetone for 10 min. Slides were rehydrated for 10 min in PBS/3% BSA and then incubated for 2 hours with primary antibodies in PBS/3% BSA. After three washes with PBS, the slides were incubated with secondary antibodies and Hoechst 33342 for 2 hours. After washing with PBS, the slides were mounted with Prolong Antifade mounting medium (Life Technologies) and left to dry overnight. Imaging was conducted on an Axio Observer7 fluorescence microscope (Zeiss) using a ×100 Oil Objective and images were processed using the ZEN 2.3 software suite. Antibodies used were mouse anti-Ty1, 1:500 (Sigma Aldrich); rat anti-HA 3F10, 1:500; goat anti-mouse Alexa 594, 1:1000 (Life Technologies); goat anti-rat Alexa 488, 1:1000 (Life Technologies); Hoechst 33342 200 nM.

### Parasite Protein Extraction and Western Blot

Cultures at around 5–10% parasitemia were pelleted by centrifugation and erythrocytes were permeabilized with 0.075% Saponin/PBS for 5 minutes on ice to release haemoglobin and parasitophorous vacuole contents. Saponin-permeabilized infected erythrocytes were washed three times in PBS and extracted in SDS sample buffer for 5 minutes at 95°C at a concentration of 1 × 10^6^ IE/µl. After removing insoluble cellular remnants such as hemozoin by centrifugation, the lysates were separated on 4–12% Bis/Tris gradient gels in 1 × MES buffer (Invitrogen). Proteins were transferred to a 0.2 µm nitrocellulose filter (Amersham) in a wet blot chamber and blocked in TBS/0.05% Tween (TBS-T) containing 5% non-fat milk powder. Primary antibodies were incubated overnight in TBS-T/5% milk at 4°C, washed with TBS-T and incubated with horseradish peroxidase (HRP) coupled secondary antibody in TBS-T/5% milk for 2 h at room temperature. Blots were washed with TBS-T and signal detection was performed by chemiluminescence using an ECL detection reagent (Merck/Millipore). Densitometry analysis was conducted using ImageJ.

### Cellular Fractionation

Parasites were freed from RBCs by lysis in 0.075% saponin in PBS. The cytoplasmic (CP) fraction was derived by incubating the parasites in lysis buffer [20 mM HEPES pH 7.8, 10 mM KCl, 1 mM EDTA, 1 mM DTT, EDTA-free protease Inhibitor (Roche)] for 30 min on ice, after which IGEPAL CA630 was added to a final concentration of 0.65%. Parasites were centrifuged at 2,500 g for 10 min at 4°C and the cytoplasmic (CP) fraction was stored at -80°C. The pellet containing the nuclei was washed once in lysis buffer and further extracted in nuclear extraction buffer [20 mM HEPES pH 7.8, 800 mM KCl, 1 mM EDTA, 1 mM DTT, EDTA-free protease Inhibitor (Roche)] for 30 min at 4°C, followed by centrifugation at 2,500 g for 30 min at 4°C and the nuclear soluble (Ns) supernatant diluted with the same volume of dilution buffer (20 mM HEPES pH 7.8, 1 mM EDTA, 1 mM DTT, 30% glycerol). The resulting pellet (P) was extracted with 2 × Laemmli buffer (P) and all fractions were analyzed by western blot on 4–12% BisTris gradient gels in 1 × MES running buffer (Invitrogen).

### Co-Immunoprecipitation

Co-IPs were conducted on mononucleosome fractions derived by Micrococcal Nuclease (MNase) treatment. Nuclei were prepared as above and resuspended in chromatin digestion buffer [50 mM Tris pH 7.4, 4 mM MgCl_2_, 1 mM CaCl_2_, EDTA-free protease inhibitor (Roche), 5 mM sodium butyrate] and incubated with MNase (20 KU, NEB) for 15 min at 37°C before the reaction was stopped by adding EDTA to a final concentration of 10 mM. The MNase fraction was derived by centrifugation at 10,000 rpm 5 min at 4°C. The MNase fraction was diluted 1/7 in RIPA buffer [50 mM Tris-HCl pH 7.4, 150 mM NaCl, 1% IGEPAL CA-630, 1 mM EDTA, EDTA-free protease inhibitor (Roche)] and protein complexes were precipitated over night at 4°C with protein G agarose beads (GE Healthcare) and mouse anti-Ty1 or rat anti-HA antibodies. The complexes were washed five times in RIPA buffer and eluted using 2 × Laemmli buffer (125 mM Tris-HCl pH 6.8, 20% glycerol, 4% SDS, 0.005% bromophenol blue, 5% beta-mercaptoethanol). Co-IP extracts were analyzed by western blot or processed for Mass Spectrometry.

### Mass Spectrometry

Co-IP extracts were separated for 5 min on a 4–12% BisTris gradient gel in MES buffer (Life Technologies). The area of the gel where the proteins had entered was excised with a clean scalpel and the gel slice was stored at −80°C before further processing for MS. For tryptic in-gel digestion, gel sections were transferred to Spin-X centrifuge tubes without membrane (Corning Costar) and centrifuged at 14,000 g to break the gel sections into smaller pieces. These were first incubated in 500 µl fixation solution (40% EtOH/10% acetic acid in ddH_2_O) for 15 min, followed by incubation with 500 µl 100% acetonitrile (ACN, LC-MS grade, Merck) for 5 min. The supernatant was discarded. Thereafter, the gel pieces were treated with 10 mM DTT in 25 mM NH_4_HCO_3_ and incubated at 56°C for 30 min. This was followed by alkylation with 55 mM iodoacetamide (Sigma Aldrich) in 25 mM NH_4_HCO_3_ for 1 h at RT in the dark. Gel pieces were then washed successively with 25 mM NH_4_HCO_3_ and 50% ACN, and finally dried in vacuo. Dry gel pieces subsequently were treated with 200 µl 50 mM NH_4_HCO_3_ containing 0.5 µg trypsin/per sample (MS grade, Serva). After incubation at 37°C overnight, peptides were extracted from the gel pieces with 200 μl ACN, followed by an incubation with 1% formic acid (FA, LC-MS grade, Sigma Aldrich) and (1:1 v/v) ACN. The supernatants were combined in a fresh Eppendorf tube, dried in a Speed Vac (Eppendorf), and reconstituted with 5 μl of 5% FA. For LC-MS/MS analysis, 4 μl of the peptide-mix spiked with 100 fmol RePLiCal (iRT-Standard; Polyquant) were loaded onto a C18-trap column (5 min, isocratic conditions A; A: 0.1% FA in ddH_2_O; 10 μl/min flow rate; YMC-TriArt: ID 300 μm; 5 mm length; 3 µm particle size) and further separated on a C18-analytical column (90 min, binary gradient 3–45% B; A: 0.1% FA and B 0.1% FA in ACN; 5 μl/min flow rate; YMC-TriArt: ID 300 μm; 15 cm length; 1.9 µm particle size). Eksigent nanoLC400 (Sciex) was used for liquid chromatography, coupled to a hybrid quadrupole time-of-flight mass spectrometer (TripleTOF5600+, Sciex). Peptides were analyzed using a TOP15 data acquisition method (DDA: TOF-MS: 400–1250 m/z, accumulation time: 250 ms and TOF-MS/MS: 200–1500 m/z, accumulation time: 75 ms). The identification of protein groups was done using the search engine Protein PilotTM Version 5.01 (Sciex) and the UniProt database PlasmodiumFalciparum3D7_08–2019. Skyline (Version 21.1.0.146; MacCoss Lab) was used for MS1 quantification. Further data processing was done in Excel (MS Office) by calculating the percentage of total peptides represented by each protein hit, and data were visualized using GraphPad Prism Version 8.3.0.

### RNA Extraction, cDNA Preparation and qPCR

RNA was isolated from *Plasmodium* culture pellets by phenol/chloroform extraction using TRIzol (Life Technologies). The RNA was further purified using the RNeasy Mini kit (Qiagen) including an on-column and an in-solution DNase I digest. Efficient gDNA removal was controlled by qPCR with primers amplifying *sbp1* (Supplementary Table S1). cDNA was synthesized using the Superscript III RT system (Invitrogen) and quantified using primers outlined in Supplementary Table S1 with SYBR Green Master Mix (Invitrogen) on an Applied Biosystems 7900HT. The level of each sequence in cDNA was calibrated relative to its level in 2.5 ng of 3D7 gDNA, and normalized to either *seryl-tRNA synthetase* or *hsp70* using the 2_-ddct_ method.

### Chromatin Immunoprecipitation

Chromatin immunoprecipitation (ChIP) was conducted as described previously (Josling et al., 2015). Briefly, culture pellets were fixed in 1% formaldehyde for 10 min at 37°C and the reaction quenched with 125 mM Glycine. Erythrocytes were lysed in 0.075% saponin in PBS, and the parasite nuclei were isolated by incubation for 30 min in lysis buffer (10 mM HEPES pH 7.9, 10 mM KCl, 0.1 mM EDTA pH 8, 0.1 mM EGTA pH 8, 1 mM DTT, 1 × cOmplete EDTA-free protease inhibitors) on ice followed by 100 strokes in a dounce homogenizer. After centrifugation, nuclei were extracted in SDS lysis buffer (1% SDS, 10 mM EDTA, 50 mM Tris pH 8.1) and the chromatin was sheared by sonication in a Bioruptor (Diagenode) to a fragment size of 200–1000 bp and diluted 1:10 in chromatin dilution buffer (0.01% SDS, 1.1% Triton X-100, 1.2 mM EDTA, 16.7 mM Tris-HCl pH 8.1, 150 mM NaCl). The diluted chromatin was precleared by incubation with BSA blocked protein G sepharose 4 Fast Flow (GE Healthcare) and precipitated over night at 4°C in the presence of BSA blocked protein G sepharose and mouse anti-Ty or rat anti-HA antibodies. The immune complexes were washed for 10 min each at 4°C in low salt immune complex wash buffer (0.1% SDS, 1% Triton X-100, 2 mM EDTA, 20 mM Tris-HCl pH 8.1, 150 mM NaCl), high salt immune complex wash buffer (0.1% SDS, 1% Triton X-100, 2 mM EDTA, 20 mM Tris-HCl pH 8.1, 500 mM NaCl), LiCl immune complex wash buffer (0.25 M LiCl, 1% NP-40, 1% deoxycholate, 1 mM EDTA, 10 mM Tris-HCl pH 8.1) and twice in TE buffer (10 mM Tris-HCl pH 8.0, 1 mM EDTA) before the complexes were eluted with elution buffer (1% SDS, 0.1 M NaHCO_3_). Chromatin was de-crosslinked in the presence of 0.5 M NaCl over night at 45°C, proteins digested with Proteinase K (NEB) for 2 h at 37°C, and the DNA was purified on MinElute columns (Qiagen) and quantified using the Qubit dsDNA HS kit (Invitrogen). ChIP qPCR was conducted using primers outlined in Supplementary Table S1 with SYBR Green Master Mix (Invitrogen) on an Applied Biosystems 7900HT fast real-time PCR system. PfBDP7Ty enrichment at each locus was calculated as % of input DNA and expressed as relative enrichment to a mouse IgG negative control.

### Read Mapping and Data Analysis

Paired end DNA libraries were prepared using the Accel-NGS^®^ 2S Plus DNA Library Kit (Swift Bioscience, 21,096) following the “Indexing by PCR” protocol. 15 ng input DNA or total chromatin-immunoprecipitated DNA was ligated to Accel-NGS^®^ 2S Truncated Adapters (28,196) and indexed by PCR using Swift Unique Dual Indexing Primers (X9096-PLATE). The indexing and library amplification step was conducted using KAPA Hifi DNA polymerase (KAPA Biosystems KK2101). PCR conditions were 98°C for 2 min (initial denaturation), followed by 12–14 cycles of 98°C for 15 s and 65°C for 2 min, with a final extension at 65°C for 5 min. Libraries were quality checked using an Agilent TapeStation and sequenced as 150 bp paired end reads on a NovaSeq 6000 instrument at the NGS Core Facility in Bonn. The resulting reads were quality checked by FastQC version v0.11.8 (http://www.bioinformatics.babraham.ac.uk/projects/fastqc/) and remaining adaptors or undefined bases were trimmed off using TrimGalore version 0.6.5 (https://github.com/FelixKrueger/TrimGalore) and Cutadapt version 2.8 (http://journal.embnet.org/index.php/embnetjournal/article/view/200) using default settings for paired reads with Illumina adaptors and a minimum resulting read length of 20 bp allowed. The resulting files were checked again using FastQC. Subsequently the reads were mapped to the *Plasmodium* genome version 28 (PlasmoDB-28_Pfalciparum3D7 available on PlasmoDB) by Bowtie2 version 2.3.5.1 (Langmead and Salzberg, 2012) using default settings for paired-end reads and transformed into bam files using Samtools version 1.12 (Li et al., 2009). The mapping quality was checked by generating a histogram representing the abundances of the possible MAPQ scores. Mapping statistics are provided as Supplementary Table S2. Bigwigfiles of the log2-ratio of ChIP samples over input samples with a quality cutoff at 28 and the option --ignoreDuplicates and --normalize Using ‘RPKM’ were then generated using Deeptools version 3.3.1 (Ramirez et al., 2016). For further analysis, the bigwig files were plotted over different gene groups using Deeptools computeMatrix version 3.3.0. Bedfiles for genomic features were generated using the R packages “rtracklayer” and “GenomicFeatures” for exons and introns. Intergenic regions were first annotated by using the coordinates between the annotated genes and subsequently divided into divergent, convergent or tandem regions depending on the gene orientation around the intergenic region. Tandem regions were further subdivided into “tandem ups” and “tandem down”, where “tandem ups” represents the 2 kb closest to the gene start, while “tandem down” represents the 2 kb closest to the gene stop.

The gene groups for heterochromatic genes in schizonts was generated using a ChIPseq dataset for H3K9me3 coverage in schizonts (3D7 background) (Jeninga et al., manuscript in preparation). Bedtools 2.28.0 multicov was used to determine H3K9me3 coverage 500 bp around every gene start to predict the probability of the gene for being heterochromatic. As we assumed that a gene could be either heterochromatic or euchromatic but nothing intermediate, we used a bivariate gaussian distribution using mixtools 1.2.0 NormalmixEM (Benaglia et al., 2009) in R-Studio, similarly as in (Fraschka et al., 2018). If the probability for being heterochromatic was > 0.99999, the gene was assumed to be heterochromatic. The remaining euchromatic genes were then sorted according to their FPKM value of a schizont RNA-Seq data set (Jeninga et al., manuscript in preparation), with 0 FPKM representing silent genes, and the other genes were divided into tertiles by expression. The tRNA genes were excluded from the silent gene group as they are not represented due to the library preparation method. The different VSA gene groups were generated by searching for either “PfEMP1”, “rifin”, “stevor”, or “2TM” in the gff file for *Plasmodium* genome version 28. Genes in regions without input coverage due to chromosomal deletions were manually excluded.

ChIPseq peaks were determined using MACS2 version 2.2.7.1 (Zhang et al., 2008) call summits with *p*-value 0.001 for PfBDP1HA::PfBDP1BirATy or *p*-value 0.01 for PfBDP7TyGlmS. Areas without input coverage were excluded and the peaks were filtered by fold change and only peaks with fold change >2 for PfBDP1HA::PfBDP1BirATy or fold change >1.6 for PfBDP7TyGlmS were included in the subsequent analyses. Bedtools closest version v2.29.2 (Quinlan and Hall, 2010) was used to determine the closest peaks either intersecting or upstream of genes. The resulting lists of genes were then intersected using Venny 2.1.0 (https://bioinfogp.cnb.csic.es/tools/venny/) and the different groups were analyzed for gene ontology enrichment using gProfiler (https://biit.cs.ut.ee/gprofiler/gost) or PlasmoDB. Bedtools intersect was used for analysis of the genomic features intersecting with the peaks. Data visualization was performed using Graph Pad Prism 8.3.0. Sequencing data are available under GEO accession number GSE186984.

### Antibodies

The following primary antibodies were used in this study: mouse anti-Ty1 clone BB2 (Sigma-Aldrich), rat anti-HA 3F10 (Sigma-Aldrich), rabbit anti-PfMC-2TM CT and rabbit anti-PfMC-2TM SC (Bachmann et al., 2015), rat anti-RIF40 (Petter et al., 2008), rabbit anti-STEVOR PfC0025c/Pf3D7_0300400 (Bachmann et al., 2015), rabbit anti-*Plasmodium* aldolase (ab207494, Abcam), rabbit anti-H3 (ab1791, Abcam), rabbit anti-HP1 and rabbit anti-H2A.Z (Petter et al., 2011).

## Results

### Pf3D7_1124300 Encodes a *Plasmodium*-Specific Protein With a Putative Bromodomain Fold Domain

**FIGURE 1:**
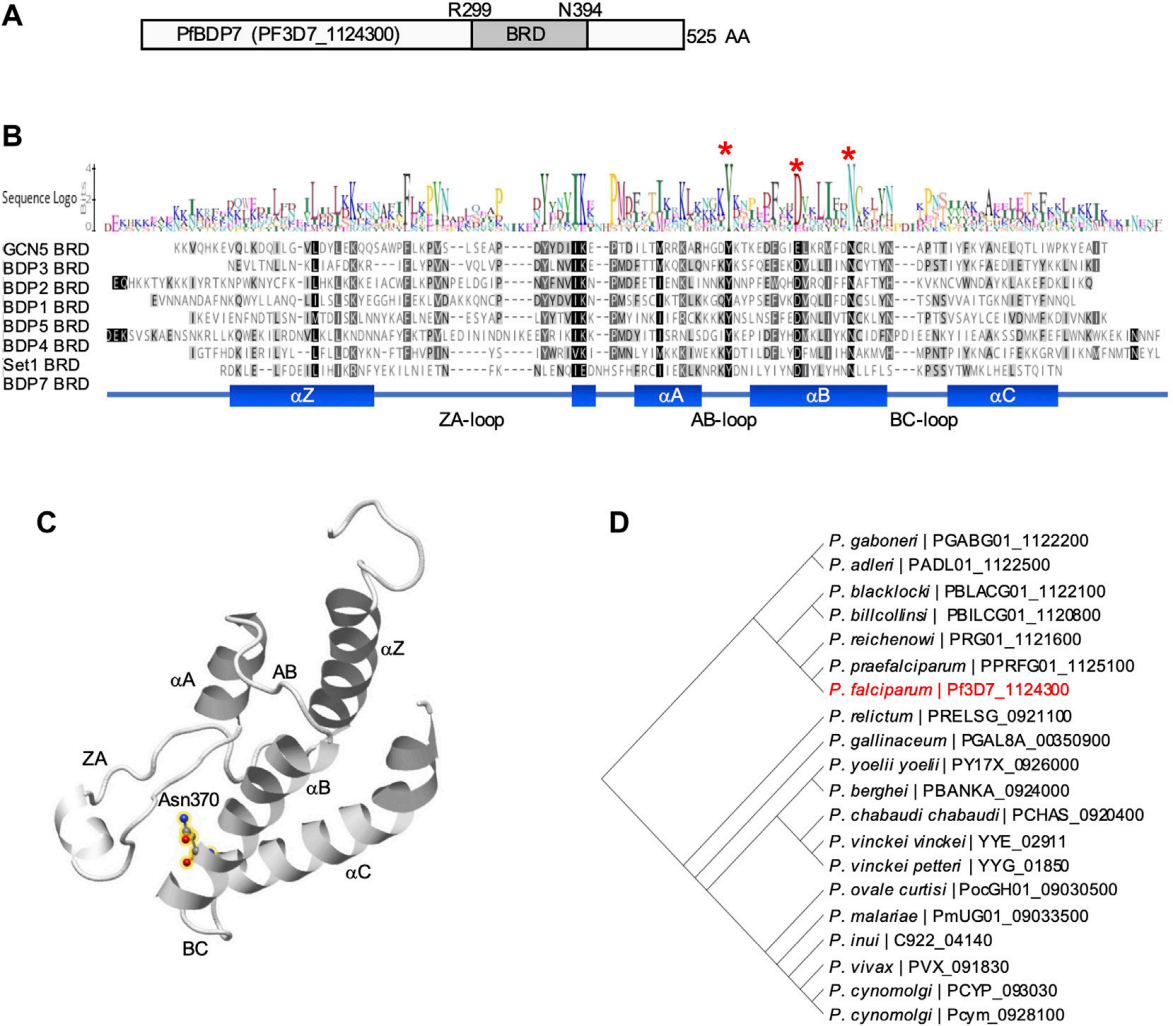
Structure prediction of PfBDP7. **(A)** Model of PfBDP7 with the putative bromodomain (BRD) from R299 to N394 highlighted in dark grey. **(B)** Alignment of *P. falciparum* bromodomains including the modelled PfBDP7 domain. Amino acids are shaded by degree of similarity. The sequence logo depicts conserved residues. Structural features (αZ, αA, αB, αC helices with inter-helical connecting loops ZA-, AB- and BC-loops) are indicated at the bottom. The conserved Y and D residues stabilizing the bromodomain fold, and the conserved N critical for Kac binding are indicated by red asterisks. **(C)** Structural model of the PfBDP7 bromodomain predicted by Phyre2. The structural features characteristic for the bromodomain fold are labelled. The conserved N370 is highlighted in yellow halos. **(D)** Phylogenetic tree of PfBDP7 orthologs identified in the *Plasmodium* genus. PF3D7_1124300 (PfBDP7) is labelled in red.

Pf3D7_1124300 encodes a 525 amino acid conserved protein of unknown function with no annotated domains. A 3D homology search using the Phyre2 protein structure prediction tool (Kelley et al., 2015) predicted that the protein contains a typical bromodomain fold structure encompassing the region between R299 and N394 (97.7% confidence). Hence, we will refer to Pf3D7_1124300 as PfBDP7 (*P. falciparum* bromodomain protein 7). The bromodomain fold comprises a left-handed four-helix bundle (αZ, αA, αB, αC), with two loops connecting the helices αZ and αA (ZA), and αB and αC (BC), respectively, which project to one side of the bundle and together form a hydrophobic pocket (Zaware and Zhou, 2019). In the αB helix, PfBDP7 encodes a conserved asparagine residue (N370), which is critical for acetylated lysine binding of bromodomains (Figure 1) (Filippakopoulos et al., 2012).

A BLAST search of the PfBDP7 sequence against the Eukaryotic Pathogen Database (www.veupathdb.org) revealed that the protein is conserved across the genus *Plasmodium.* The only orthologs outside of the *Plasmodium* genus were predicted in the closely related apicomplexan parasites *Hepatocystis sp.* and *Theileria equi*. However, closer inspection of multiple sequence alignments (Clustal Omega, https://www.ebi.ac.uk/Tools/msa/clustalo/) showed that the putative bromodomain was only present in *Plasmodium* sequences, pointing towards a specific function of PfBDP7 in *Plasmodium* parasites. Across all *Plasmodium* species, PfBDP7 was highly conserved in sequence, particularly from E307 to Y525, comprising the putative bromodomain and the C-terminal part. All sequences contained the functionally important Asn residue in the αB helix, as well as a conserved Tyr residue in the AB loop that typically forms a salt bridge with an Asp in the αB helix to stabilize the fold (Supplementary Figure S1).

### PfBDP7 Tightly Interacts With the BDP1/2 Core Complex

**FIGURE 2:**
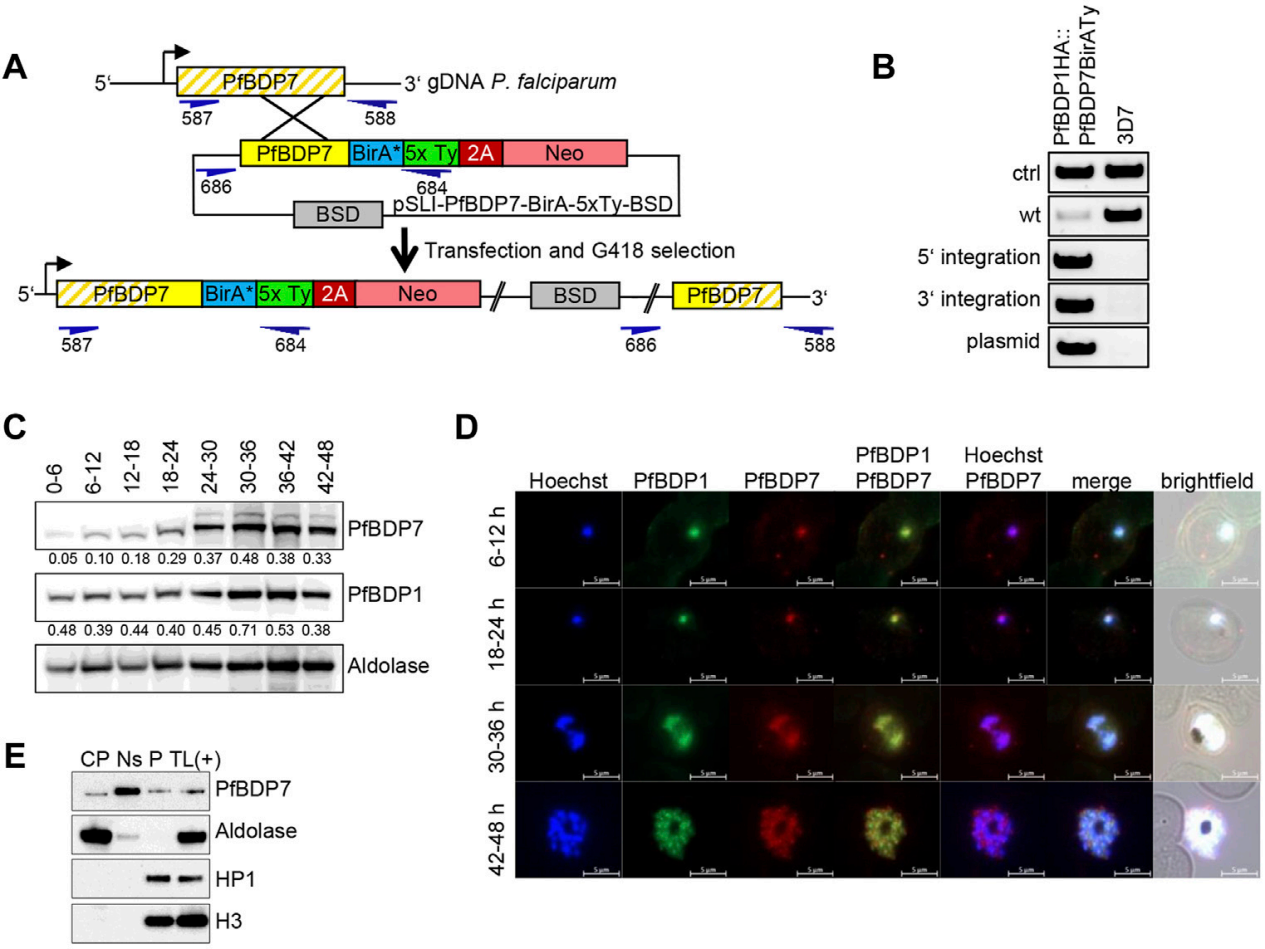
PfBDP7 expression during the *P. falciparum* asexual life cycle. **(A)** Scheme of SLI strategy for modification of the PfBDP7 genomic locus by integration of the BirA* biotin ligase and 5xTy sequences fused to a 2A skip peptide and the neomycin (Neo) selection cassette. Primer positions for diagnostic PCR are indicated in dark blue, with numbers according to Supplementary Table S1. **(B)** Diagnostic PCR screen for plasmid integration of the double transgenic parasite line PfBDP1HA::PfBDP7BirATy (wt = wild type, ctrl = control). **(C)** Western blot analysis of PfBDP7BirATy expression across the 48 h asexual parasite life cycle relative to PfBDP1HA and aldolase as a loading control. Synchronized parasite lysates were prepared in 6 h intervals. Relative intensities compared to aldolase were determined by densitometry and are indicated underneath each blot. **(D)** Immunofluorescence analysis of PfBDP1HA::PfBDP7BirATy double transgenic parasites using antibodies detecting the HA tag fused to PfBDP1 or the Ty tag fused to PfBDP7. Hoechst 33342 was used for DNA staining. The parasite age post invasion of the parasites is indicated on the left (h). Scale bars, 5 µm. **(E)** Cellular fractionation of PfBDP7Ty parasites in cytoplasmic (CP), salt soluble nuclear (Ns) and pellet (P) fractions. A total lysate of PfBDP7Ty parasites as positive control [TL (+)] was analyzed alongside. The Western Blots were probed with antibodies detecting Ty, aldolase, HP1 (heterochromatin protein 1) or H3 (histone 3).

PfBDP7 was identified in close association with PfBDP1 and PfBDP2 in previous studies by mass spectrometric analyses, and the complex was dubbed the “BDP1/2 core complex” (Josling et al., 2015; Hoeijmakers et al., 2019). We aimed to confirm the functional interaction with PfBDP1 and to investigate the two proteins relative to each other to study their individual contributions to gene expression. We transfected a PfBDP1HA parasite line (Josling et al., 2015) with a plasmid allowing for selection linked integration (SLI) (Birnbaum et al., 2017) to endogenously tag PfBDP7 with five copies of the Ty1 epitope tag. To facilitate future proteomic analysis of the complex by BioID, we also incorporated the promiscuous biotin ligase BirA* (Roux et al., 2013) into the locus (Figure 2A), yielding the parasite line PfBDP1HA::PfBDP7BirATy. The correct manipulation of the PfBDP7 locus was confirmed by diagnostic PCR (Figure 2B). Western Blot analysis showed that PfBDP7 and PfBDP1 were co-expressed throughout the entire *P. falciparum* life cycle (Figure 2C). Peak expression of PfBDP1 and PfBDP7 relative to the cytoplasmic control protein aldolase occurred in late trophozoites at 30–36 hpi, consistent with increased chromatin content due to nuclear replication during schizogony. A band of minor intensity running at a slightly higher molecular weight than the expected 104 kDa for PfBDP7BirATy fusion protein was also evident and may be due to posttranslational modifications of the protein. Indirect immunofluorescence analysis (IFA) demonstrated that PfBDP1 and PfBDP7 colocalized in the nucleus throughout the IDC (Figure 2D). To further characterize the physical association of PfBDP7 with the nuclear compartment, we performed cellular fractionation assays in schizont stage parasites (Figure 2E). Western Blot analysis showed that the bulk of PfBDP7 was present in the high salt soluble nuclear fraction, with minor enrichment in the salt insoluble chromatin fraction in the pellet. In contrast, heterochromatin protein 1 (HP1) and histone H3 were tightly associated with the salt insoluble chromatin fraction (Figure 2E). This supports the nuclear localization of PfBDP7 detected by IFA and identifies PfBDP7 as a salt-soluble chromatin associated factor.

**FIGURE 3:**
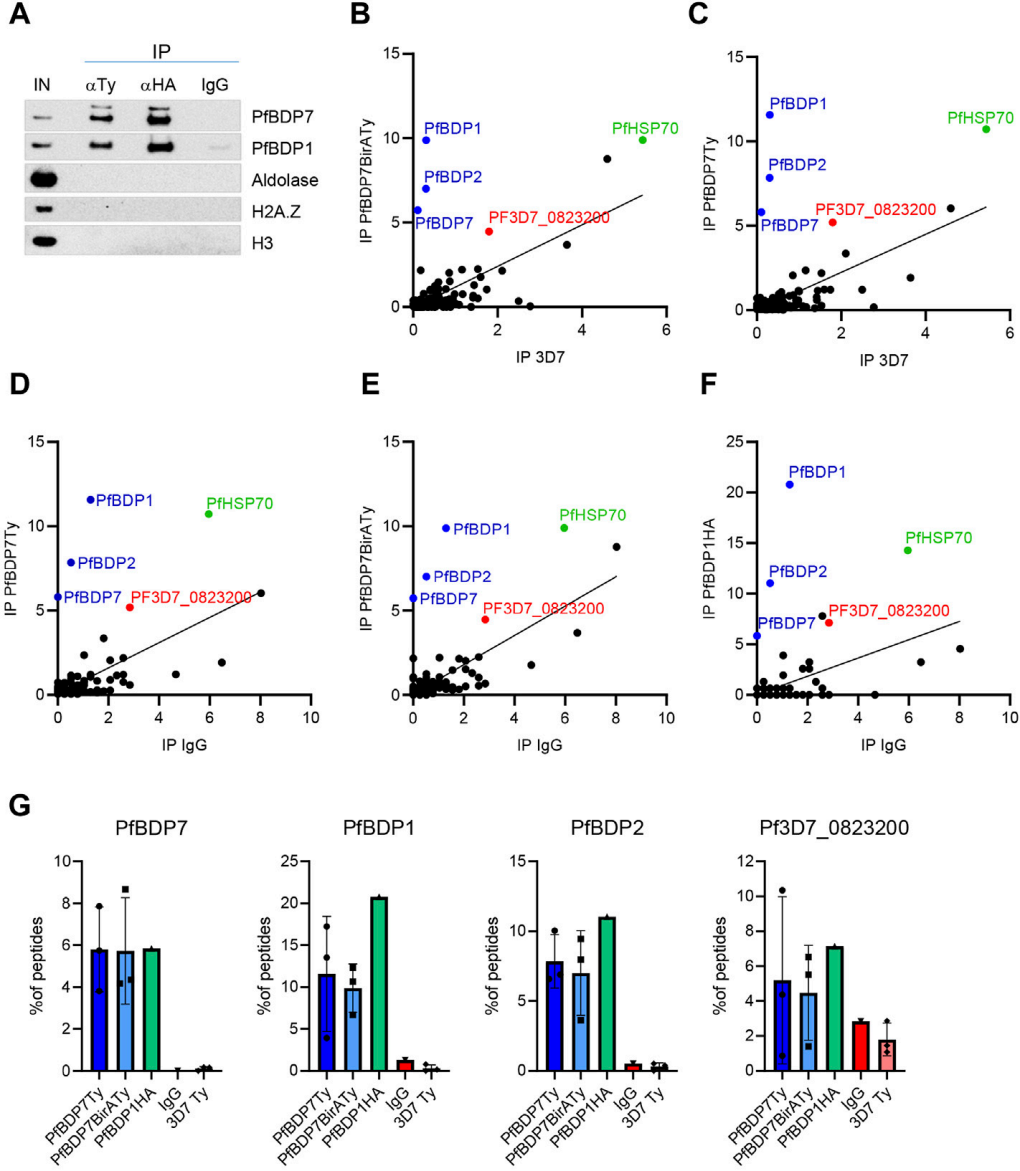
Identification of PfBDP7 interaction partners. **(A)** Co-Immunoprecipitation of PfBDP7BirATy and PfBDP1HA from PfBDP1HA::PfBDP7BirATy parasites demonstrates that the two proteins are part of the same protein complex. The immunoprecipitation (IP) from micrococcal nuclease treated chromatin (input, IN) was performed with mouse anti-Ty, rat anti-HA or IgG antibodies as negative control. **(B–F)** PfBDP7 or PfBDP1 associated proteins were isolated by Co-IP and identified by Mass Spectrometry. Enrichment of peptides was calculated as % peptide counts of all peptides identified in each IP, biological replicates were averaged (*N* = 1-3, see Supplementary Table S3) and plotted against the negative controls. IP 3D7: anti-Ty IP in 3D7 wt parasites, IP IgG: non-immune IgG on PfBDP7Ty_GlmS parasites. **(G)** Enrichment of PfBDP7, PfBDP1, PfBDP2 and Pf3D7_0823200 peptides from individual IPs.

Several previous studies have identified PfBDP7 in complex with PfBDP1 and PfBDP2 (Hoeijmakers et al., 2019; Josling et al., 2015; Toenhake et al., 2018). However, chromatin proteins frequently associate with functionally distinct sub-complexes of variable composition (Hoeijmakers et al., 2019). To identify additional binding partners of PfBDP7, we performed reciprocal co-immunoprecipitation (co-IP) assays on micrococcal nuclease (MNase) treated nuclear extracts of schizont stage PfBDP1HA::PfBDP7BirATy parasites using anti-HA and anti-Ty antibodies, as well as non-immune IgG as negative control. Western Blot analysis showed specific pulldown of PfBDP1 with PfBDP7 and vice versa, confirming that both proteins interact tightly with each other (Figure 3A). Of note, we could not detect any nucleosomes associated with the complex under these experimental conditions, which is evident from the absence of H3 or H2A.Z after co-IP (Figure 3A). The material precipitated with anti-HA and anti-Ty was then analyzed by liquid chromatography-mass spectrometry (LC-MS) (Figures 3B–G, Supplementary Table S3). In addition, we also performed co-IP experiments with anti-Ty antibodies on nuclear extracts from wild type 3D7 parasites as a negative control, and from a second transgenic parasite line expressing PfBDP7Ty in a 3D7 background (PfBDP7Ty_GlmS; see next section for details). All comparisons confirmed the intimate interaction of PfBDP7 with PfBDP1 and PfBDP2. Apart from this tripartite complex, only the predicted RNA-binding protein Pf3D7_0823200 was consistently enriched. This protein is annotated as an orthologue of *P. berghei* UIS12, which has been functionally implicated in sexual differentiation of gametocytes and sporogony (Muller et al., 2021).

### Low Levels of PfBDP7 Are Sufficient to Maintain Parasite Viability

We attempted to disrupt the PfBDP7 locus in NF54 parasites by truncating the gene using the pSLI-TGD strategy (Birnbaum et al., 2017). Parasites that carried the episomal plasmid could be recovered from transfection, but three independent attempts to select for integration by G418 treatment failed, indicating that PfBDP7 fulfils an essential function in *P. falciparum*. This is in agreement with high essentiality scores predicted for PfBDP7 using a global transposon mutagenesis approach (Zhang et al., 2018).

**FIGURE 4:**
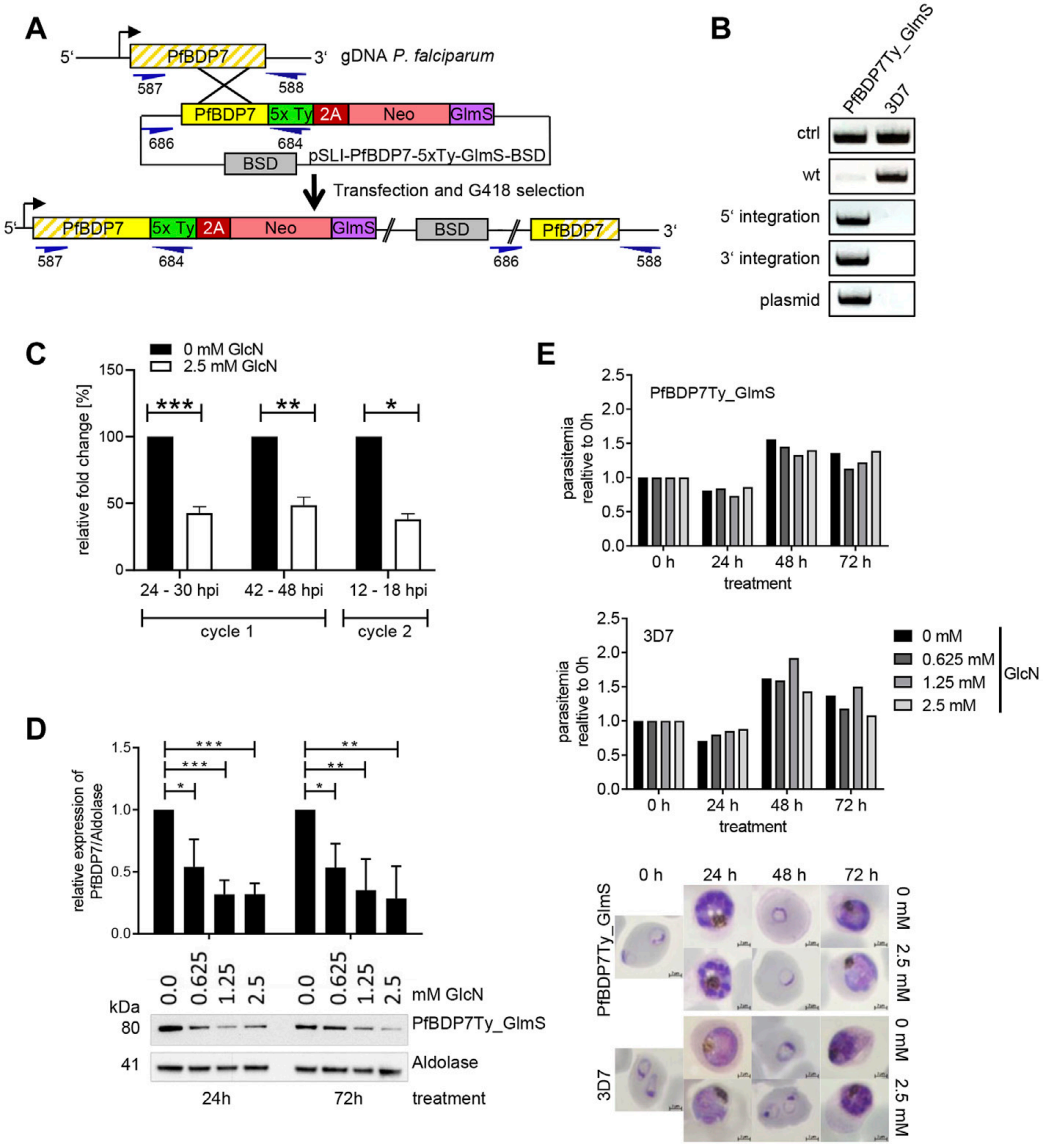
Conditional knock down of PfBDP7 does not affect parasite growth. **(A)** Scheme of SLI strategy for modification of the PfBDP7 genomic locus by integration of 5 × Ty epitope tags and a GlmS ribozyme downstream of the 2A skip peptide and the neomycin (Neo) selection cassette. Primer positions for diagnostic PCR are indicated in dark blue, with numbers according to Supplementary Table S1. **(B)** Diagnostic PCR screen for plasmid integration in the PfBDP7Ty_GlmS parasite line and (wt = wild type, ctrl = control primers targeting the *pfs16* gene). **(C)** qPCR analysis demonstrates efficient mRNA knock down in parasites treated with 2.5 mM glucosamine over two IDCs. Expression data were normalized to seryl-tRNA synthetase and the enrichment relative to untreated parasites was calculated. Mean and SD of *N* = 3, unpaired *t*-test. **(D)** Glucosamine treatment significantly reduces PfBDP7 protein expression. Tightly synchronized ring stage PfBDP7Ty_GlmS parasites (0–6 hpi) were treated for 24 or 72 h with increasing concentrations of glucosamine (GlcN) ranging from 0 to 2.5 mM, and protein expression was monitored by western blot with a Ty and a aldolase antibodies. Densitometry results are mean and SD of *N* = 3 replicates, unpaired *t*-test. **(E)** Parasite growth of PfBDP7Ty_GlmS and 3D7 control parasites treated with increasing concentrations of GlcN was examined after 24, 48, and 72 h by flow cytometry. After 24 h, the cultures were split 1:5 to prevent overgrowth. Parasite development was monitored in parallel by Giemsa staining. Representative images of cultures treated with 0 or 2.5 mM GlcN are shown.

To enable the functional investigation of PfBDP7, we generated the parasite line PfBDP7Ty_GlmS, in which endogenous PfBDP7 was fused to five copies of the Ty epitope tag. Moreover, we integrated a glucosamine-6-phosphate riboswitch ribozyme (GlmS ribozyme) into the 3’ UTR following the 2A-Neomycin selection cassette to facilitate conditional knock down of PfBDP7 (Figure 4A). Correct integration was verified by diagnostic PCR (Figure 4B). Treatment of ring stage parasite cultures with 2.5 mM glucosamine resulted in significant downregulation of PfBDP7 mRNA in trophozoites (24–30 hpi), schizonts (42–48 hpi) and ring stage parasites of the following cycle (12–18 hpi) (Figure 4C). Moreover, increasing concentrations of glucosamine from 0.625 to 2.5 mM induced a significant reduction of PfBDP7Ty on the protein level (Figure 4D, Supplementary Figure S2A). However, even at the highest concentration there was no significant effect on parasite growth over two consecutive parasite cycles. Parasites progressed normally through ring, trophozoite and schizont stages and showed no alterations in morphology or replication rate (Figure 4E). To monitor whether PfBDP7 depletion would affect parasite growth at a later time point, parasites treated with 0 or 2.5 mM glucosamine were followed for a total of four replication cycles, but SYBR green I-based detection of parasite DNA showed no significant difference between treated and untreated cultures (Supplementary Figure S2B). This indicated that the residual level of PfBDP7 protein was still enough to maintain parasite viability, or that PfBDP7 was dispensable for normal growth.

### PfBDP1 and PfBDP7 Are Important For the Repression of Small Variant Surface Antigens

**FIGURE 5:**
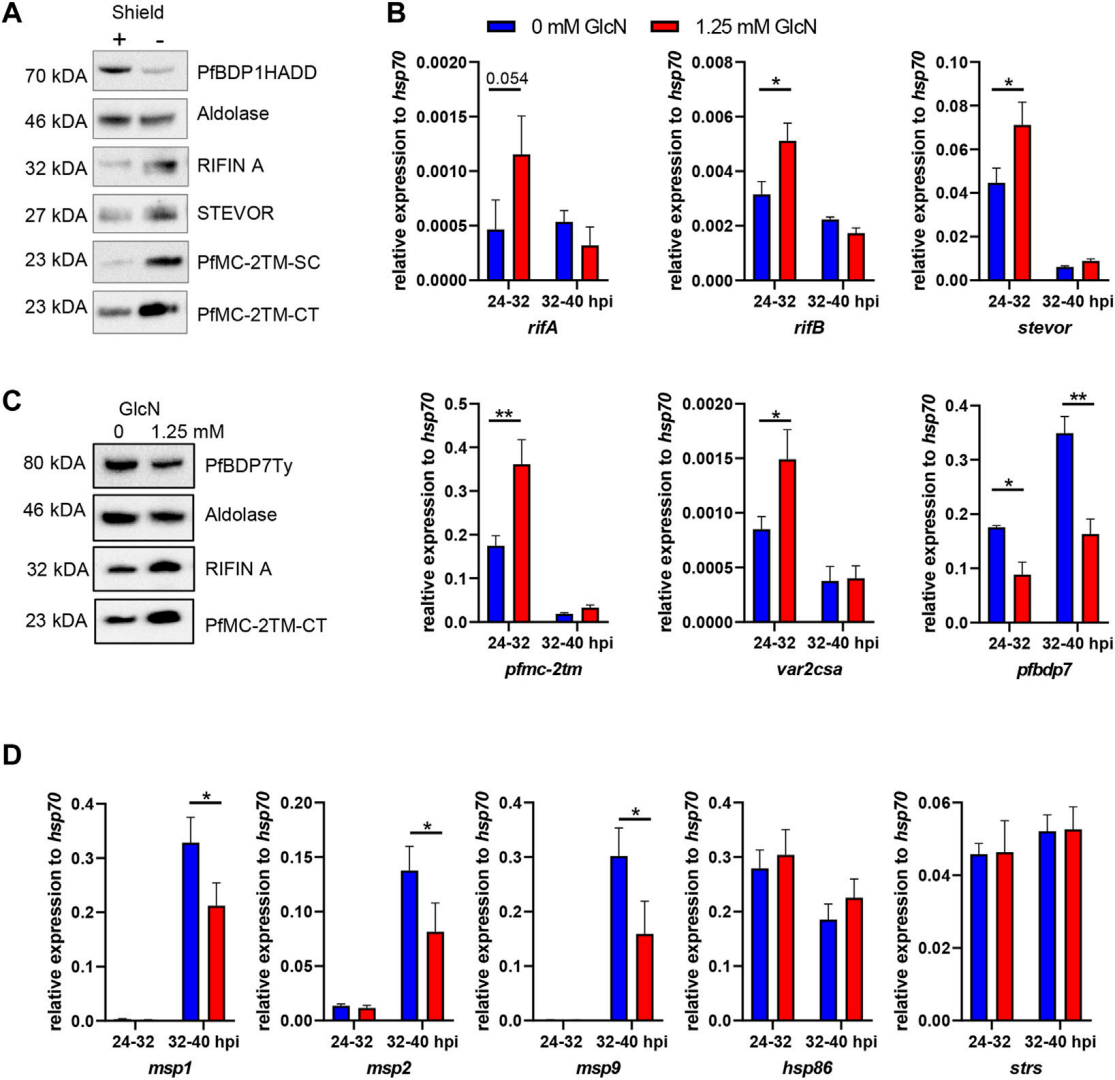
Conditional depletion of PfBDP1 or PfBDP7 results in increased variant surface antigen expression and decreased invasion gene expression. **(A)** Western Blot of PfBDP1HADD trophozoite stage parasite lysates cultivated for 24 h in the presence (+, wt) or absence (−, KD) of Shield1. Antibodies were directed against A-type RIFIN variant RIF40 (Petter et al., 2008), STEVOR variant PF3D7_0300400 (Bachmann et al., 2015); the semi-conserved (SC) or the C-terminus (CT) of PfMC-2TM (Bachmann et al., 2015). One representative example of *N* = 3 biological replicates is shown. **(B)** qPCR analysis of PfBDP7Ty_GlmS parasites cultivated with 0 or 1.25 mM glucosamine (GlcN) (mean and SD of *N* = 3). Glucosamine was added to synchronized ring stage parasites (0–8 hpi) and RNA was harvested in the next cycle at 24–32 hpi and 32–40 hpi. cDNA was quantified with degenerate primers amplifying conserved motifs in members of the, *rifA*, *rifB*, *stevor*, or *pfmc-2tm* families; or specific primers amplifying *var2csa* and *pfbdp7* and signals were normalised to *hsp70*. *t*-test, *p < 0.05, **p < 0.01. **(C)** Western Blot probing small variant surface antigen expression in lysates of trophozoite stage PfBDP7Ty_GlmS parasites cultivated with 0 or 1.25 mM GlcN. Treatment commenced in ring stage parasites and lysates were prepared after 24 h **(D)** qPCR analysis of PfBDP7Ty_GlmS parasites with primers specific for the invasion genes *msp1*, *msp2* and *msp9* as well as for *hsp86* and *seryl-tRNA synthetase (strs)* as additional controls.

Previously, we demonstrated that PfBDP1 has an essential function in regulating invasion gene expression among other genes, but conditional knock down also resulted in an upregulation of genes coding for small VSA such as the RIFIN, STEVOR and PfMC2TM families on the transcriptional level (Josling et al., 2015). To test whether the upregulation of small VSA gene transcription was paralleled by an increased abundance of VSA proteins, we induced PfBDP1 knock down in our PfBDP1HADD parasite line at the ring stage and performed Western Blot analyses 24 h later using antibodies directed against semi-conserved epitopes of each VSA family, which have been previously demonstrated to recognize multiple variants across different parasite isolates (Bachmann et al., 2015). Indeed, all small VSA families showed significant upregulation on the protein level, confirming that PfBDP1 is important for VSA repression (Figure 5A).

Antigenic variation is critical for parasite virulence *in vivo*, but represents a non-essential process in *P. falciparum in vitro* culture, thus allowing us to study the functional impact of PfBDP7 on small VSA expression. To monitor whether PfBDP7 cooperates with PfBDP1 in the regulation of antigenic variation, we measured small VSA transcripts by qPCR in PfBDP7Ty-GlmS parasites treated with 0 or 1.25 mM glucosamine. RNA was prepared at 24–32 h post invasion, when peak expression of small VSAs has been observed (Bachmann et al., 2012) and at 32–40 h post invasion, when invasion genes are transcribed. Consistent with a contribution of PfBDP7 to the repression of small VSAs, the *pfmc2tm, rifB and stevor* gene families were significantly upregulated in glucosamine treated parasites, as was the gene coding for the chondroitin sulfate A-binding PfEMP1 variant *var2csa* (Figure 5B). Western Blot analysis confirmed that RIFIN and PfMC-2TM proteins were increased in PfBDP7Ty knock down parasites (Figure 5C). Interestingly, several invasion genes that we tested in parallel by qPCR were significantly down-regulated after PfBDP7 knock down, albeit only by less than 50%, which may explain why no effect on parasite growth was observed (Figure 5D). Taking these findings together, we concluded that PfBDP7 and PfBDP1 cooperate in both invasion gene expression as well as in VSA repression, but may also contribute to the regulation of other genes.

### PfBDP1 and PfBDP7 Jointly Bind to Regulatory Regions

**FIGURE 6:**
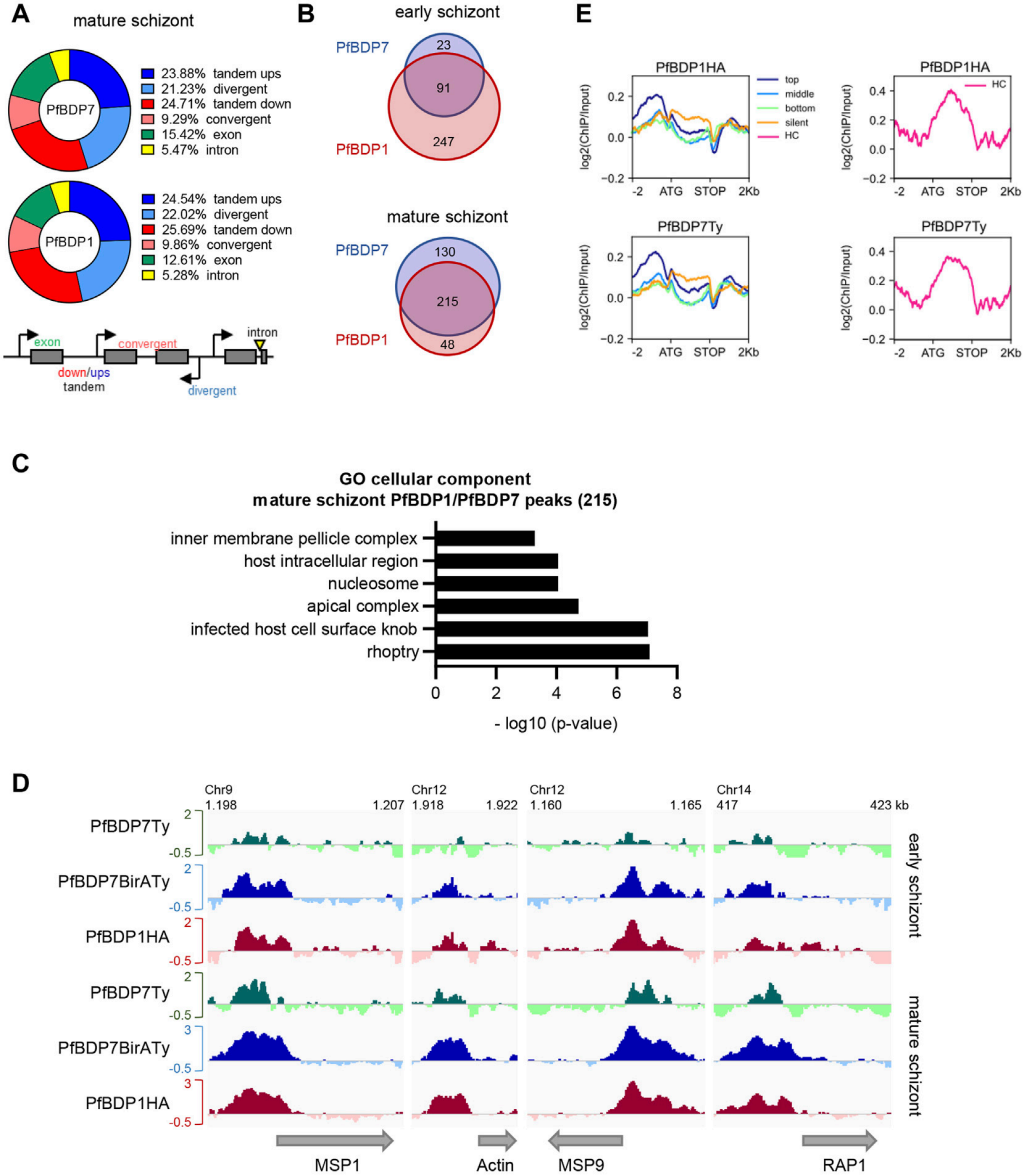
PfBDP7 interacts with PfBDP1 across the genome. Chromatin immunoprecipitation was performed on PfBDP7Ty_GlmS and PfBDP1HA::PfBDP7BirATy parasites in early and mature schizont stage parasites. **(A)** Chart depicting % of PfBDP7 and PfBDP1 peaks localized in the specified genomic features in PfBDP1HA::PfBDP7BirATy mature schizont stage parasites. Ups = upstream, down = downstream. The relative orientation of genetic elements is indicated in the scheme. **(B)** Venn diagram visualizing overlap of genes closest to PfBDP1 and PfBDP7 peaks in early and mature PfBDP1HA::PfBDP7BirATy schizont stage parasites. **(C)** Gene ontology (GO) analysis of genes closest to common PfBDP1 and PfBDP7 peaks in mature schizonts. The most significant, non-redundant GO terms concerning “cellular component” are presented. **(D)** Enrichment profiles [log 2 (ChIP/Input)] of PfBDP7 and PfBDP1 in selected invasion genes. Green: PfBDP7Ty (from PfBDP7Ty_GlmS parasite ChIP); blue: PfBDP7BirATy and red: PfBDP1HA (from PfBDP1HA::PfBDP7BirATy parasite ChIP). **(E)** Line plots of log2 (ChIP/Input) ratios for PfBDP7 and PfBDP1 in mature PfBDP1HA::PfBDP7BirATy schizont stage parasites. Euchromatin associated genes were ranked into top, medium, bottom or non-expressed genes (silent) by schizont stage expression. Heterochromatin (HC) associated genes were plotted separately (right panels, pink lines).

We previously mapped genome wide binding sites of PfBDP1 by chromatin immunoprecipitation (ChIPseq) in schizont stage parasites, which showed that PfBDP1 is associated with invasion gene promoters (Josling et al., 2015). To investigate the genome wide profile of PfBDP7 relative to PfBDP1, we performed cross-linked ChIP experiments in PfBDP1HA::PfBDP7BirATy early (34–42 hpi) and mature (40–48 hpi) schizont stage parasites using anti-HA and anti-Ty antibodies. In addition, we also ChIPed PfBDP7 from PfBDP7Ty_GlmS parasites. The majority of identified PfBDP1 and PfBDP7 peaks were localized in intergenic regions either upstream of genes (with tandem or divergent orientation) or downstream of genes (with tandem or convergent orientation) at both stages (Figure 6A, Supplementary Figure S3A).

**FIGURE 7:**
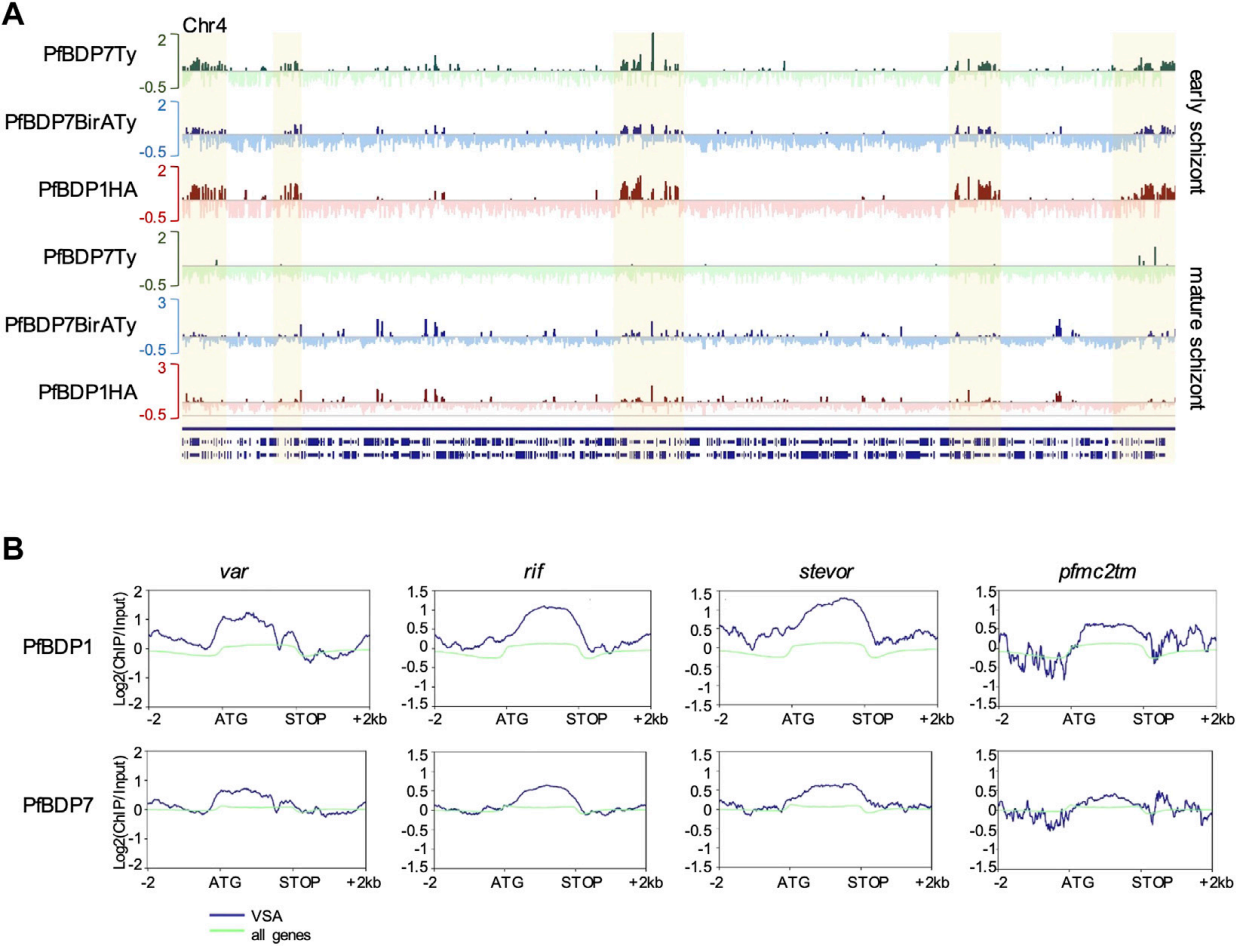
PfBDP7 and PfBDP1 are enriched across VSA gene bodies in early schizonts. **(A)** Log2 (ChIP/input) enrichment profiles of PfBDP7 and PfBDP1 in early schizonts across chromosome 4 depicts accumulation of ChIP signals in heterochromatic regions (highlighted in yellow) containing VSA genes. Green: PfBDP7Ty_Glms; blue: PfBDP7BirATy; red: PfBDP1HA. **(B)** Line plots of average PfBDP1 and PfBDP7 enrichment across VSA families *var*, *rif*, *stevor* and *pfmc-2tm* (blue) and all genes (green) in PfBDP1HA::PfBDP7BirATy early schizonts.

Analysis of the genes that were located in proximity to the PfBDP1 and PfBDP7 peaks in PfBDP1HA::PfBDP7BirATy parasites showed a large overlap in both early and mature schizonts (Figure 6B, Supplementary Table S4). Consistent with our earlier findings, many genes associated with PfBDP1 and PfBDP7 in mature schizonts were related to host cell invasion [gene ontology (GO) analysis: inner membrane pellicle complex, apical complex, rhoptry] (Figure 6C, Supplementary Table S4). Closer inspection of the enrichment profiles at several invasion genes clearly demonstrated that both factors commonly occupy promoters of invasion genes (Figure 6D). In addition, both PfBDP1 and PfBDP7 enrichment was strongest in the promoters of the top schizont stage transcribed genes, implicating both bromodomain proteins in the transcriptional activation of schizont-expressed genes (Figure 6E). However, we also noted that, particularly in early schizonts, PfBDP1 and PfBDP7 both accumulated in heterochromatic regions including subtelomeric and internal clusters (Figures 6E, 7A, Supplementary Table S4). In these regions, all of the VSA families are located. Indeed, all VSA families displayed an accumulation of both PfBDP1 and PfBDP7 along their gene bodies (Figure 7, Supplementary Figures S3C,D); and genes showing peaks of the two bromodomain proteins were linked to GO terms such as adhesion to symbiont or host, host cell plasma membrane, and infected host cell surface knob in early schizonts (Supplementary Figure S3B, Supplementary Table S4). Accumulation of PfBDP1 in the heterochromatin compartment was also evident in an independent ChIPseq experiment of NF54::PfBDP1HA schizonts (Supplementary Figure S3E). Interestingly, genes that were associated with heterochromatin but did not belong to the major VSA families (*var*, *rif, stevor*, *pfmc-2tm*) showed intermediate occupancy of PfBDP1 and PfBDP7 (Supplementary Figure S3F). Thus, while PfBDP1 and PfBDP7 are implicated in activation of host cell invasion in mature schizont stage parasites, they also appear to contribute to the silencing of VSA families by directly interacting with heterochromatin.

Several ApiAP2 transcription factors have been implicated in the biology of subtelomeric regions where VSAs are primarily located, including SIP2 (Flueck et al., 2010), AP2exp (Martins et al., 2017) and AP2tel (Sierra-Miranda et al., 2017). SIP2 is enriched in SPE2 motifs which are present in clusters upstream of subtelomeric *var* genes; however, its exact function in telomere biology and VSA regulation remains speculative (Flueck et al., 2010). Disruption of AP2exp function by truncation has been reported to result in increased *pfmc-2tm*, *rifin* and *stevor* expression, but *var* genes, which carry the putative AP2exp binding motif 1500 bp upstream, remained unaffected (Martins et al., 2017). AP2tel binds through its AP2 domain to telomere repeats and, thus, may influence subtelomeric heterochromatin, but to date no functional studies have been conducted to support a role in VSA expression (Sierra-Miranda et al., 2017). To find out whether PfBDP1 and PfBDP7 also contribute to the regulation of transcription factors that have previously been linked to VSA expression and telomere biology, we inspected the chromatin profile along the genes coding for the ApiAP2 factors SIP2, AP2exp and AP2tel. Only the *sip2* gene promoter showed peaks in early and mature schizonts, and only the *sip2* transcription level was affected by PfBDP7 knock down (Supplementary Figure S4). Thus, PfBDP1- and PfBDP7-dependent regulation of SIP2 may also indirectly contribute to heterochromatin structure and regulation of VSA expression.

## Discussion

Understanding the divergent mechanisms underlying gene expression in the human malaria parasite *P. falciparum* is an important pillar in designing novel antimalarial intervention strategies. Here, we functionally characterized the chromatin-associated protein PfBDP7, which is specific to the genus of *Plasmodium*. We demonstrate that in early schizonts, PfBDP7 and PfBDP1 cause repression of heterochromatin-associated genes, i.e. small VSA genes, whereas in mature schizonts, PfBDP7 cooperates with PfBDP1 in the activation of invasion related genes. PfBDP7 contains an unannotated bromodomain fold, indicating that this protein may bind to acetylated lysine residues and thereby increases the specificity of chromatin recruitment of the BDP1/BDP2 core complex to genomic target sites. The bromodomain fold was also identified in a search of the AlphaFold protein structure database (https://alphafold.ebi.ac.uk/), which is currently regarded as the top-ranked protein structure prediction method as rated by CASP14 (14th Community Wide Experiment on the Critical Assessment of Techniques for Protein Structure Prediction) (Jumper et al., 2021). Interestingly, AlphaFold also predicts the highly conserved C-terminal part to fold into a domain consisting of three alpha helices connected by a loop containing two anti-parallel beta strands. Elucidating the exact biochemical function of both domains in future investigations will give further insight into the mechanistic basis of PfBDP7-mediated transcriptional regulation and may explain the unexpected finding of PfBDP7 association with subtelomeric and central heterochromatin domains in early schizonts. Indeed, bromodomain proteins frequently contain multiple protein-protein interaction modules, which can fine tune the specificity of chromatin targeting, for example through combining acetyl-binding bromodomains with methyl-reader PHD (plant homeodomain) fingers that may interact with other modifications in nucleosomes in a multivalent fashion, or with post-translational modifications on other chromatin associated proteins (Fujisawa and Filippakopoulos, 2017). For instance, the mammalian tandem PHD/bromodomain containing protein TRIM28/TIF1B/KAP1 functions as a transcriptional repressor of pluripotency genes by modulating heterochromatin structure through the interaction with phosphorylated HP1, thereby driving cellular differentiation processes (Cammas et al., 2002; Gehrmann et al., 2019; Qin et al., 2021). Importantly, the function of TRIM28 in heterochromatin assembly and maintenance is regulated by SUMOylation of TRIM28 (Ivanov et al., 2007), demonstrating that post-translational modifications of bromodomain proteins can influence both their chromatin targeting and their function in gene regulation. This also suggests a potential mechanism through which PfBDP1 and PfBDP7 could associate with both heterochromatic and euchromatic nucleosomes in a developmentally regulated fashion. In this respect, it is worth noting that both PfBDP1 and PfBDP7 carry multiple stage-specific acetylation and phosphorylation sites, which may modulate their chromatin association (Treeck et al., 2011; Pease et al., 2013; Cobbold et al., 2016; Ganter et al., 2017). Thus, it will be of interest to further identify and functionally investigate posttranslational modifications of these bromodomain proteins in future studies.

Conditional knock down of both PfBPD1 or PfBDP7 led to an increase in the expression of the small VSA families on both RNA and protein level, including RIFIN, STEVOR and PfMC-2TM families, functionally implicating the two bromodomain proteins in gene silencing. This was consistent with our previous observation of increased transcription of *rifin*, *stevor* and *pfmc2tm* genes upon conditional PfBDP1 knock down (Josling et al., 2015). Interestingly, *var* genes seemed to be affected to a lesser extent by PfBDP1 knock down, although we also detected increased transcription of *var2csa* after PfBDP7 knock down in the present study. In support of a direct repressive function of PfBDP1 and PfBDP7 in VSA regulation, we found that particularly in early schizonts the two proteins were enriched across gene bodies of genes coding for VSAs. At first glance, the enrichment of an acetyl-binding bromodomain protein complex in the generally deacetylated heterochromatin compartment appears to be counter-intuitive. However, PfBDP1 was also identified in pull-down assays of telomere repeats together with the ApiAP2 family DNA binding protein AP2tel (Sierra-Miranda et al., 2017), suggesting that PfBDP1 and PfBDP7 may bind to as yet unidentified acetylated sites of heterochromatic nucleosomes or heterochromatin associated proteins. Several interactions between mammalian BET family bromodomain proteins and acetylated transcription factors have been documented that are critical for gene regulation [reviewed in (Fujisawa and Filippakopoulos, 2017)]. Indeed, many members of the *P. falciparum* ApiAP2 family of DNA binding proteins including the heterochromatin associated factors SIP2, AP2tel, and AP2exp also carry acetylations (Cobbold et al., 2016), through which they may be directly bound by bromodomains. In addition, DNA/RNA binding proteins of the ALBA (acetylation lowers binding affinity) family are associated with heterochromatin in *P. falciparum* and have been detected in complex with PfBDP1, representing an additional putative non-histone target for PfBDP1 and PfBDP7 at heterochromatic sites (Chene et al., 2012; Goyal et al., 2012; Josling et al., 2015). In our present mass spectrometric analysis, we further found the putative RNA binding protein Pf3D7_0823200 consistently enriched together with the core complex of PfBDP1, PfBDP2 and PfBDP7. While the *P. berghei* orthologue of Pf3D7_0823200, UIS12, is non-essential in blood-stage parasites but has a role in transmission (Muller et al., 2021), its function in *P. falciparum* remains unknown.

Recently, it was shown that parasites that carry a deletion of the bromodomain of the histone acetyltransferase PfGCN5, lose the mutually exclusive expression of the *var* genes and upregulate multiple *var* genes in a single cell at the same time, implicating PfGCN5 in *var* gene silencing (Miao et al., 2021). Consistent with a direct association with VSA genes, PfGCN5 was also mapped by ChIPseq to the heterochromatic compartment in trophozoites, providing another example of a heterochromatin associated *P. falciparum* bromodomain protein (Rawat et al., 2021). Interestingly, PfGCN5 KO also showed a similar phenotype as PfBDP1 depletion regarding invasion gene regulation, thus functionally linking the two bromodomain complexes with each other (Miao et al., 2021). As GCN5 is known to mediate both histone and non-histone protein acetylations (Downey, 2021), it is tempting to speculate that PfGCN5 may catalyse the acetylations to which PfBDP1 and PfBDP7 bind in both euchromatin and heterochromatin. However, this remains to be experimentally validated.

In summary, our study unravels a dual function of the two bromodomain proteins PfBDP1 and PfBDP7 in the repression of heterochromatin-regulated gene families and in the activation of invasion-related, and potentially also other target genes, which demands further mechanistic investigations. It remains to be shown whether the bromodomain PfBDP2, which is tightly associated with PfBDP1 and PfBDP7 (Josling et al., 2015; Hoeijmakers et al., 2019), also associates with heterochromatin, or whether functionally distinct subcomplexes exist. In addition, the impact of PfBDP1 and PfBDP7 depletion on heterochromatin formation and its maintenance will be of interest. Knockout of the bromodomain-containing *Saccharomyces pombe* AAA-ATPase Abo1 resulted in a global decrease in nucleosomal occupancy as well as a transcriptional de-repression and loss of heterochromatic silencing (Gal et al., 2016), which was associated with reduction of H3K9me3 in heterochromatic regions (Dong et al., 2020). Both PfBDP7 and PfBDP1 contain additional domains of unknown function, which may carry catalytic activities directly related to chromatin remodelling or indirectly by serving as adaptors for the recruitment of the chromatin remodelling and transcriptional machineries. In conclusion, our study adds a new piece in the puzzle of chromatin-based gene regulation in malaria parasites and opens up new avenues to investigate the *Plasmodium*-specific bromodomain proteins PfBDP1 and PfBDP7 as targets for novel anti-malaria interventions.

## Data Availability

The datasets presented in this study can be found in online repositories. The names of the repository/repositories and accession number(s) can be found below: Sequencing data are available under GEO accession number GSE186984.

## Supplementary Material

SUPPLEMENTARY FIGURE S1 Multiple sequence alignment of PfBDP7 orthologues from different *Plasmodium* strains. (A) Conserved bromodomain (BRD) highlighting the conserved Y and D residues stabilizing the bromodomain fold, and the conserved N critical for Kac binding (red asterisks). (B) Conserved C-terminal domain.

SUPPLEMENTARY FIGURE S2 PfBDP7 knock down leads to reduced PfBDP7 expression but has no effect on parasite growth. (A) Assessment of PfBDP7Ty expression in parasites treated for 4 days with various concentrations of glucosamine by IFA. Green: anti-Ty; blue Hoechst. (B) SYBR green I based growth assay over 4 consecutive growth cycles.

SUPPLEMENTARY FIGURE S3 PfBDP7 and PfBDP1 enrichment in early schizonts. (A) Chart depicting localization of PfBDP7 and PfBDP1 peaks in specified genomic features in early schizont stage of PfBDP1HA::PfBDP7BirATy parasites. Ups = upstream, down = downstream. The relative orientation of genetic elements is indicated in the scheme in Figure 6A. (B) Gene ontology (GO) analysis of genes closest to common PfBDP1 and PfBDP7 peaks in early schizonts. The most significant, non-redundant GO terms concerning “biological process” and “cellular component” are presented. (C) Log2 ChIP/Input ratios of PfBDP7 and PfBDP1 in a central heterochromatin cluster on chromosome 7 (highlighted in yellow). ChIPs were performed on PfBDP7Ty_GlmS parasites (green) or PfBDP1HA::PfBDP7BirATy parasites (blue: PfBDP7, red: PfBDP1). (D) Validation of PfBDP7Ty enrichment in PfBDP7Ty_GlmS parasites in an independent ChIP experiment by qPCR for *msp1*, *msp9*, and *sip2* intergenic regions (ups, blue) as well as open reading frames (orf) of several *var*, *rifin*, and *stevor* genes (red, dots represent data from individual genes, error bars represent SD of the mean. The orf of *msp1* was analyzed as a locus showing low PfBDP7 enrichment (black). (E) Line plots of log2 (ChIP/Input) ratios for PfBDP1HA in NF54::PfBDP1HA schizont stage parasites from an independent ChIPseq experiment. Euchromatin associated genes were ranked into top, medium, bottom or non-expressed genes (silent) by schizont stage expression. Heterochromatin (HC) associated genes were plotted separately (right panel, pink lines). (F) Line plots of log2 (ChIP/Input) ratios for PfBDP7 and PfBDP1 in immature PfBDP1HA::PfBDP7BirATy schizont stage parasites. Heterochromatin associated genes were grouped as VSA including (*var*, *rif*, *stevor* and pfmc-2tm) and non-VSA (heterochromatic genes other than VSA).

SUPPLEMENTARY FIGURE S4 PfBDP7 and PfBDP1 bind to the promoter of SIP2 and contribute to its regulation. (A) Log2 transformed ChIP/Input ratio of PfBDP7Ty and PfBDP1HA/PfBDP7BirATy parasites using anti-Ty and anti-HA antibodies. (B) Transcription of ApiAP2 family members in PfBDP7Ty_GlmS parasites treated with 0 or 1.25 mM glucosamine (GlcN). N = 3.

SUPPLEMENTARY TABLE S1 Primer sequences.

SUPPLEMENTARY TABLE S2 ChIPseq Mapping Statistics.

SUPPLEMENTARY TABLE S3 Mass Spectrometry.

SUPPLEMENTARY TABLE S4 ChIP enriched genes and GO analysis.
