# Supplementary figures and images for: The Putative Bromodomain Protein PfBDP7 of the Human Malaria Parasite Plasmodium Falciparum Cooperates With PfBDP1 in the Silencing of Variant Surface Antigen Expression
Source: Front Cell Dev Biol. 2022 Apr 12;10:816558. doi: 10.3389/fcell.2022.816558 (PMC9039026; doi:10.3389/fcell.2022.816558)

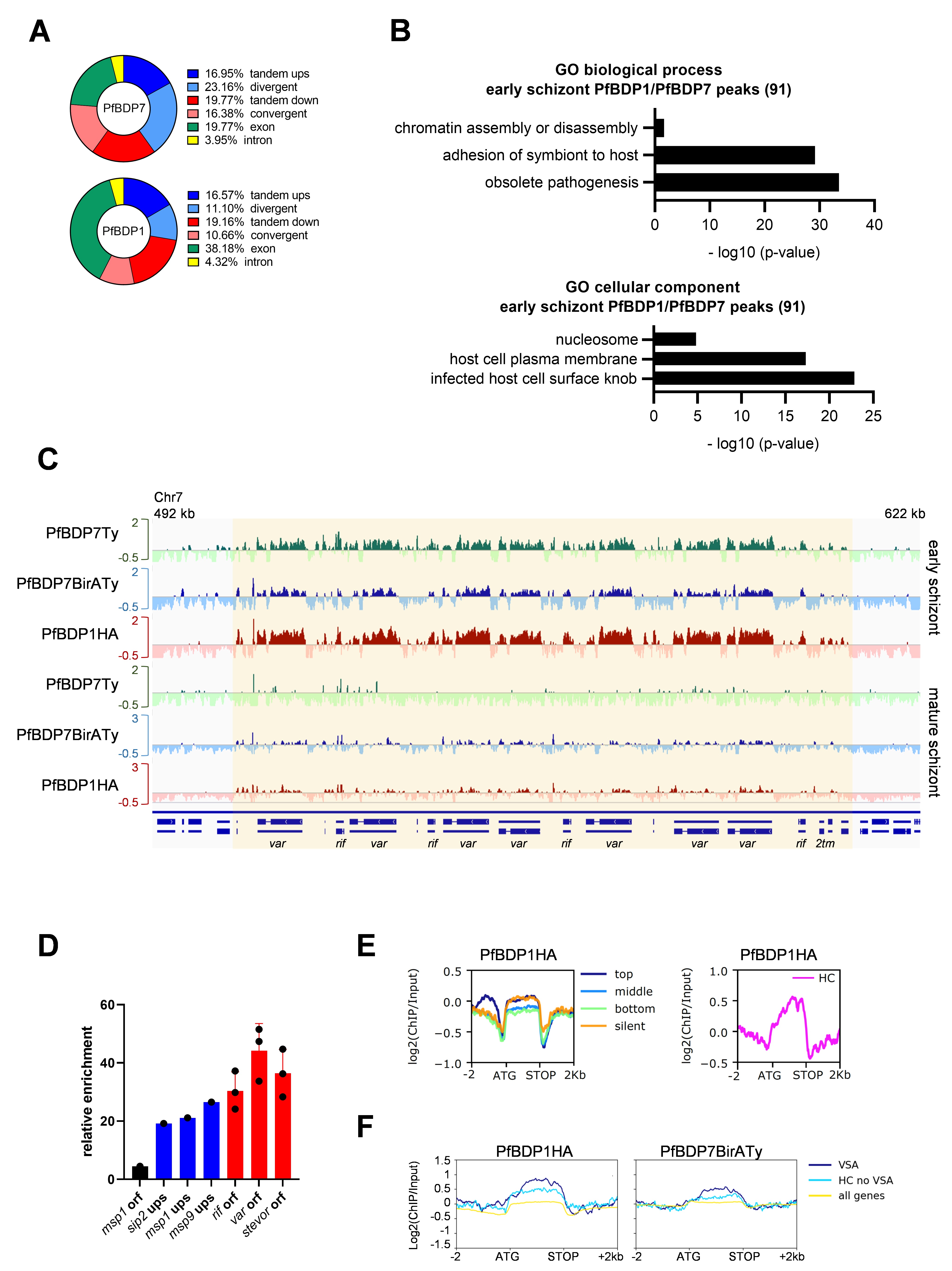

Supplement: Supplementary file 2 [file Image3.JPEG]

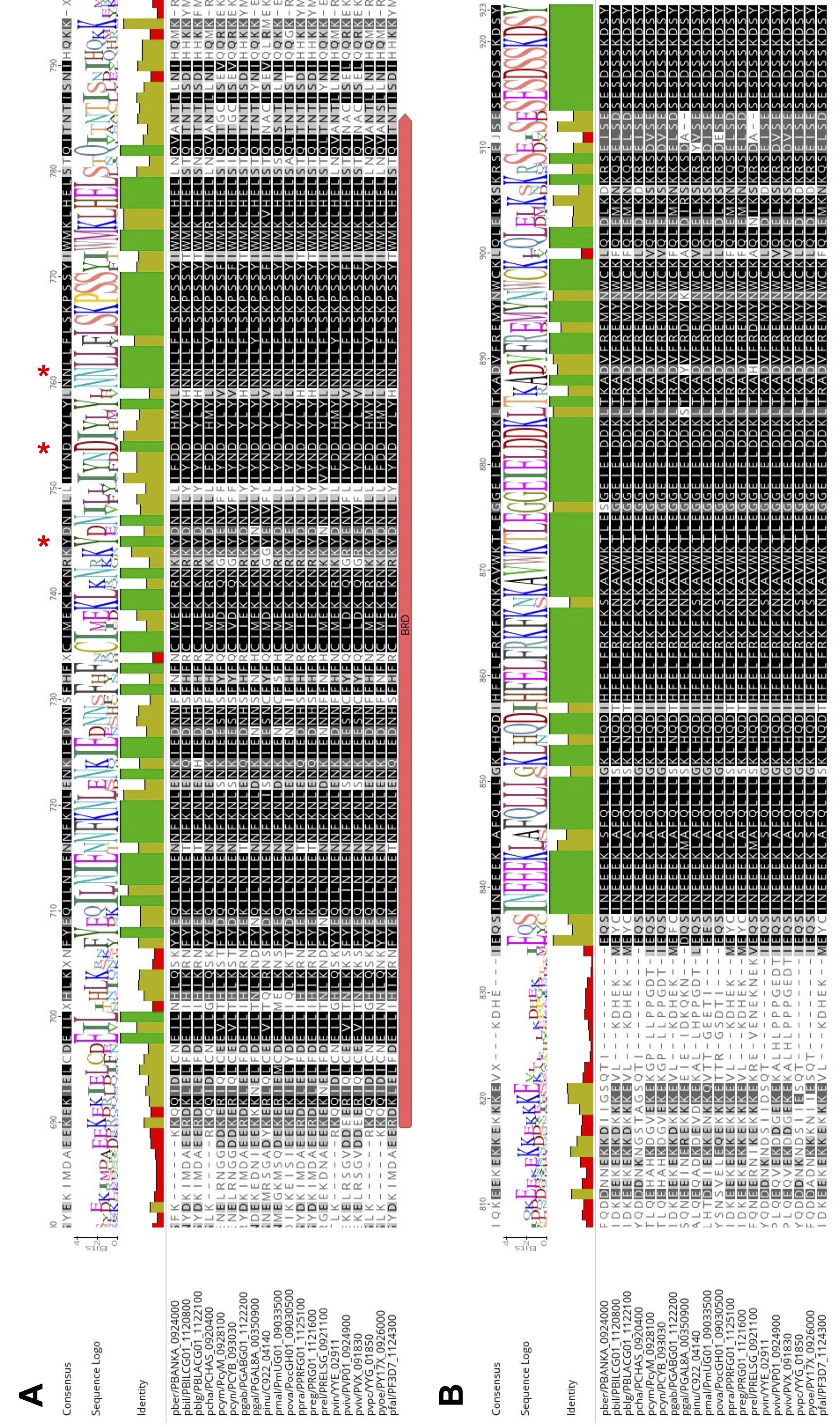

Supplement: Supplementary file 5 [file Image1.JPEG]

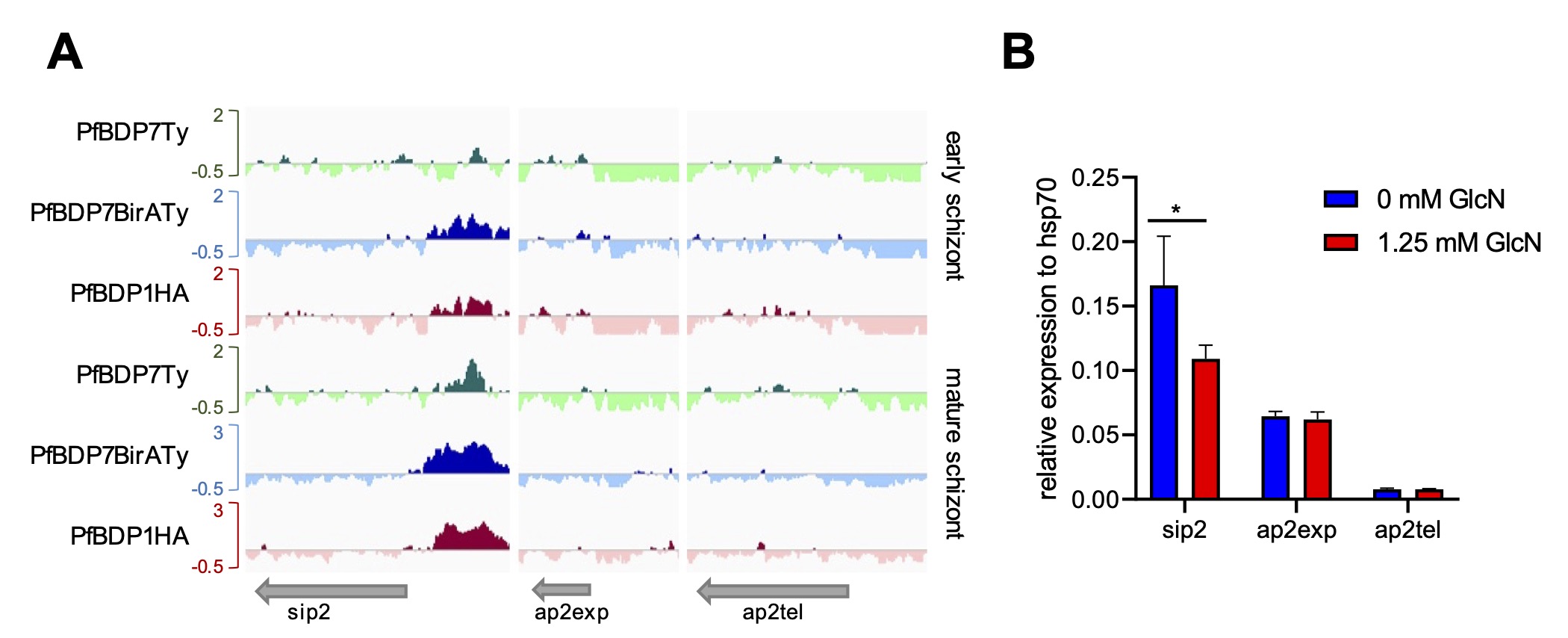

Supplement: Supplementary file 6 [file Image4.JPEG]

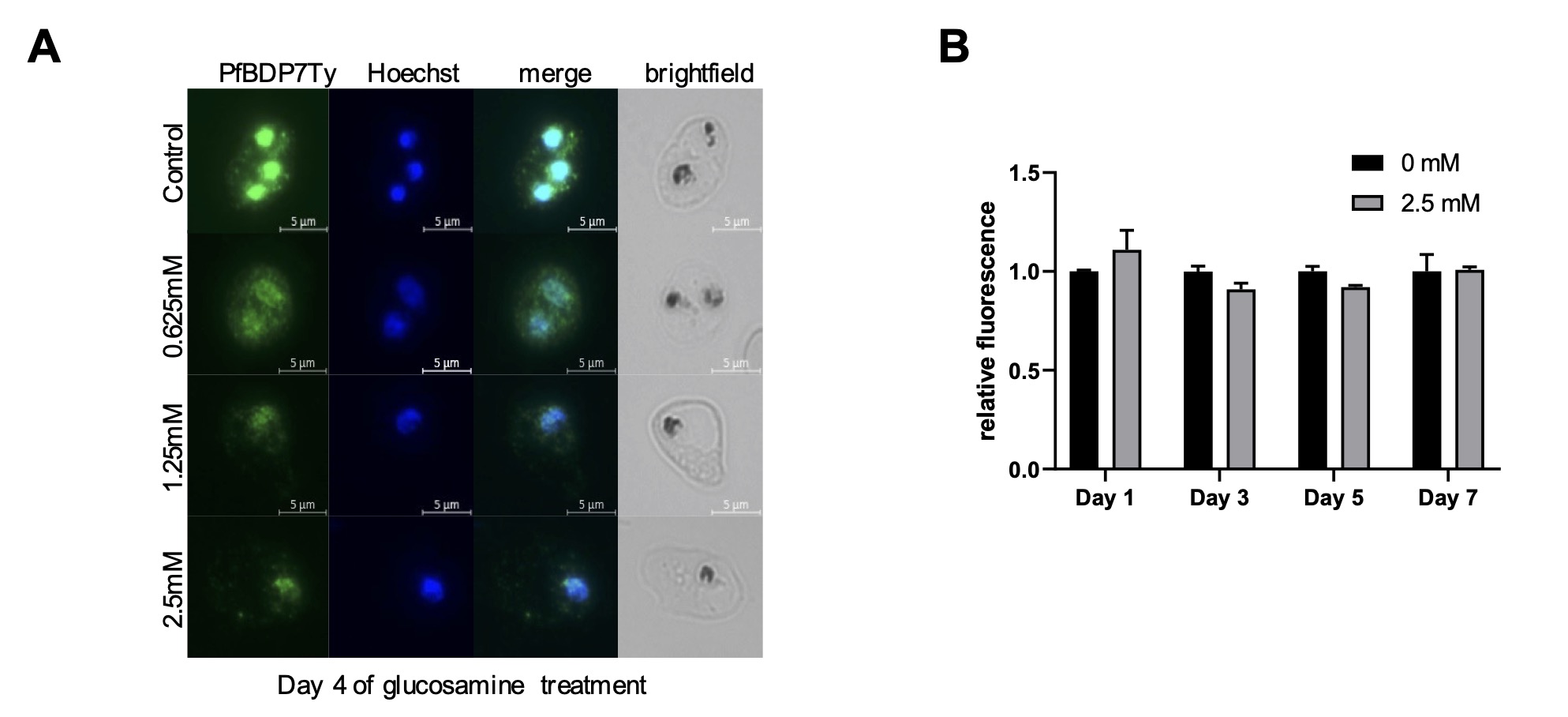

Supplement: Supplementary file 7 [file Image2.JPEG]
