# Supplementary material for: The Putative Bromodomain Protein PfBDP7 of the Human Malaria Parasite Plasmodium Falciparum Cooperates With PfBDP1 in the Silencing of Variant Surface Antigen Expression
Source: Front Cell Dev Biol. 2022 Apr 12;10:816558. doi: 10.3389/fcell.2022.816558 (PMC9039026; doi:10.3389/fcell.2022.816558)
Supplement: Supplementary file 4 [file Table1.DOCX]

**Supplementary Table S1: Oligonucleotides used in this study**

| **Purpose** | **Primer** | **Sequence (5’ – 3’)** | **Target** |
| --- | --- | --- | --- |
| Cloning | 443 | tttcccgggTGGATGCAGAAGAAAGAGAT | XmaI PfBDP7 for |
| Cloning | 451 | tttgctagcGTAAGAATCCTTTGATGAATCGCT | NheI PfBDP7 rev |
| Cloning | 484 | tttACTAGTGGAGAAGGAAGAGGAAGTTT | SpeI SLI_2A_Neo for |
| Cloning | 485 | tttACTAGTCCTAGGACGCGTTTAGAAGAACTCGTCAAGAAGGC | SpeI SLI_2A_Neo MluI/AvrII rev |
| Cloning | 486 | tttACGCGTTAATTATAGCGCCCGAACTAAGC | MluI Glms for |
| Cloning | 487 | tttCCTAGGAGATCATGTGATTTCTCTTTGT | AvrII Glms rev |
| Cloning | 488 | tttGCTAGCATGAAAGATAATACAGTACCA | NheI BirA for |
| Cloning | 489 | tttGCTAGCTTTTTCAGCTGATCTTAATGA | NheI BirA rev |
| Diagnostic PCR | 587 | GATATTGAAAAGGTACGAAGTTATGA | PfBDP7 5' int for |
| Diagnostic PCR | 588 | TGGTCTCTTCTTTTCCTCCA | PfBDP7 3' int rev |
| Diagnostic PCR | 684 | ACTAGTGTCAAGTGGATCCTG | Ty rev |
| Diagnostic PCR | 686 | TTTTCTTCCCACATTTCGA | Hsp86 promoter for |
| Control | 496 | ACATACCAAATTCTTCTTTTCCA | pfs16 for |
| Control | 497 | AGAATCATCTCCTTCGTCTCC | pfs16 rev |
| qPCR | 98 | CAGCAAGCGAAACAACTGAAGA | msp1 for |
| qPCR | 99 | GATTGGGATAATGTGTGTGTGGA | msp1 rev |
| qPCR | 357 | ACCCAAAGTTAAAGCACAACCA | msp9 for |
| qPCR | 358 | TCTTCCGTTGGTTCAACACCT | msp9 rev |
| qPCR | 25 | TCCTGTACCTTTATTCTCTGGTG | msp2 for |
| qPCR | 26 | CAAGCTGAAAATTCTGCTCC | msp2 rev |
| qPCR | 1023 | TCGAGGTACCATTAAATTCACGT | sip2 for |
| qPCR | 1024 | ACATTCCATCCAACTGCCAA | sip2 rev |
| qPCR | 1029 | TCCCAGTACATCTTGCCAAAA | ap2tel for |
| qPCR | 1030 | GTTCGGCGGACAATTCTACC | ap2tel rev |
| qPCR | 1015 | GTAGGAAGACCTAGAGGTGCTAGTTCA | ap2exp for |
| qPCR | 1016 | GCCACGCACACATACGCTTATT | ap2exp rev |
| qPCR | 763 | ACATGAACTCTCGACGCAAA | pfbdp7 for |
| qPCR | 764 | GAAGCAACTGGAAAGCCAAC | pfbdp rev |
| qPCR | 563 | CCW SAA ATG AAA GAA GTD ATG | rifA deg for |
| qPCR | 564 | CTT TRT CRC ATT KWT CTT TAC A | rifA deg rev |
| qPCR | 565 | CGA CAA RCN TCA CAA MGW TT | rifB deg for |
| qPCR | 566 | CAC CTC CTA RCC CAC ACC CAC ACH TAA G | rifB deg rev |
| qPCR | 569 | GGA ACA TAC AAA YYA TCA TAC CAT AAT | pfmc-2tm deg for |
| qPCR | 570 | CAA TAT ATT CKT TAA GGY ATT TCC | pfmc-2tm deg rev |
| qPCR | 567 | CCR CAT TAT CAT AAT GAY CC | stevor deg for |
| qPCR | 568 | CTA CTA CWT CTT TCA ATT GTT YAT ATG G | stevor deg rev |
| qPCR | 655 | TGGTGATGGTACTGCTGGAT | var2csa for |
| qPCR | 656 | TTTATTTTCGGCAGCATTTG | var2csa rev |
| qPCR | 31 | GGTATTCCACCTGCACCAAGAA | hsp70 for |
| qPCR | 32 | CAGCCGTAACGTTTAAGATACCGT | hsp70 rev |
| qPCR | 653 | AAGTAGCAGGTCATCGTGGTT | seryl-tRNA-S for |
| qPCR | 654 | TTCGGCACATTCTTCCATAA | seryl-tRNA-S rev |
| ChIPqPCR | 571 | TGTCTAACCATTTGTATATGTTG | sip2 ups for |
| ChIPqPCR | 572 | GCAGAAAGACAAAAACTTCT | sip2 ups rev |
| ChIPqPCR | 413 | ACTTATGTGTGTAAAAGTGTTTTTGT | msp1 ups for |
| ChIPqPCR | 414 | TTGAAATAAAAAGTTTAACGGATTGT | msp1 ups rev |
| ChIPqPCR | 98 | CAGCAAGCGAAACAACTGAAGA | msp1 orf for |
| ChIPqPCR | 99 | GATTGGGATAATGTGTGTGTGGA | msp1 orf rev |
| ChIPqPCR | 355 | TTTTTCTTTCTTTTTTTCTCATAAACG | msp9 ups for |
| ChIPqPCR | 356 | AAACAAAAGGAGAGTCACAAAA | msp9 ups rev |
| ChIPqPCR | 718 | CACACCACATAAAACTAACCCC | PF3D7_0413200 rifin orf for |
| ChIPqPCR | 719 | GTGCATACAATTCACATTCGCA | PF3D7_0413200 rifin orf rev |
| ChIPqPCR | 742 | TTTGTGCATGTGGGTTAGGG | PF3D7_1254500 rifin orf for |
| ChIPqPCR | 743 | ACCGGCTTTAGTAACAGCAA | PF3D7_1254500 rifin orf rev |
| ChIPqPCR | 150 | TTGCTGTAAATGCGTGGAAAG | PF3D7_1200500 rifin orf for |
| ChIPqPCR | 151 | CCGCTTCCTTAGCCGCTATAAT | PF3D7_1200500 rifin orf rev |
| ChIPqPCR | 746 | AGTCTCATTCAAAACAACACCA | PF3D7_1254600 stevorf orf for |
| ChIPqPCR | 747 | TATGGATTTGCGTTTGTGCT | PF3D7_1254600 stevorf orf rev |
| ChIPqPCR | 738 | ACAAAACAATCATTACAGTTTCAA | PF3D7_0617600 stevor orf for |
| ChIPqPCR | 739 | TCGTTGTTCTTTGTGTGTTGT | PF3D7_0617600 stevor orf rev |
| ChIPqPCR | 261 | CACCGACGCACCTTACACCT | PF3D7_1200400 var orf for |
| ChIPqPCR | 262 | GCGCTTGTGGTGACTTACCA | PF3D7_1200400 var orf rev |
| ChIPqPCR | 41F | GGTGTCAAGGCAGCTAATGA | PF3D7_0800200 var orf for |
| ChIPqPCR | 41R | TATGTCCTGCGCTATTTTGC | PF3D7_0800200 var orf rev |
| ChIPqPCR | 655 | TGGTGATGGTACTGCTGGAT | PF3D7_1200600 var2csa orf for |
| ChIPqPCR | 656 | TTTATTTTCGGCAGCATTTG | PF3D7_1200600 var2csa orf rev |
